# Development of a Telescoped Flow Process for the Safe and Effective Generation of Propargylic Amines

**DOI:** 10.3390/molecules24203658

**Published:** 2019-10-10

**Authors:** Kian Donnelly, Huan Zhang, Marcus Baumann

**Affiliations:** School of Chemistry, University College Dublin, Science Centre South, Belfield, Dublin 4, Ireland; kian.donnelly@ucdconnect.ie (K.D.); huan.zhang@ucdconnect.ie (H.Z.)

**Keywords:** azides, flow chemistry, continuous process, propargylic amines, building block synthesis.

## Abstract

Propargylic amines are important multifunctional building blocks that are frequently exploited in the synthesis of privileged heterocyclic entities. Herein we report on a novel flow process that achieves the safe and effective on-demand synthesis of propargylic amines in a telescoped manner. This process minimizes exposure to hazardous azide intermediates and renders a streamlined route into these building blocks. The value of this approach is demonstrated by the rapid generation of a small selection of drug-like thiazolines that result from a high-yielding reaction cascade between propargylic amines with different aryl isothiocyanates.

## 1. Introduction

**Scheme 1:**
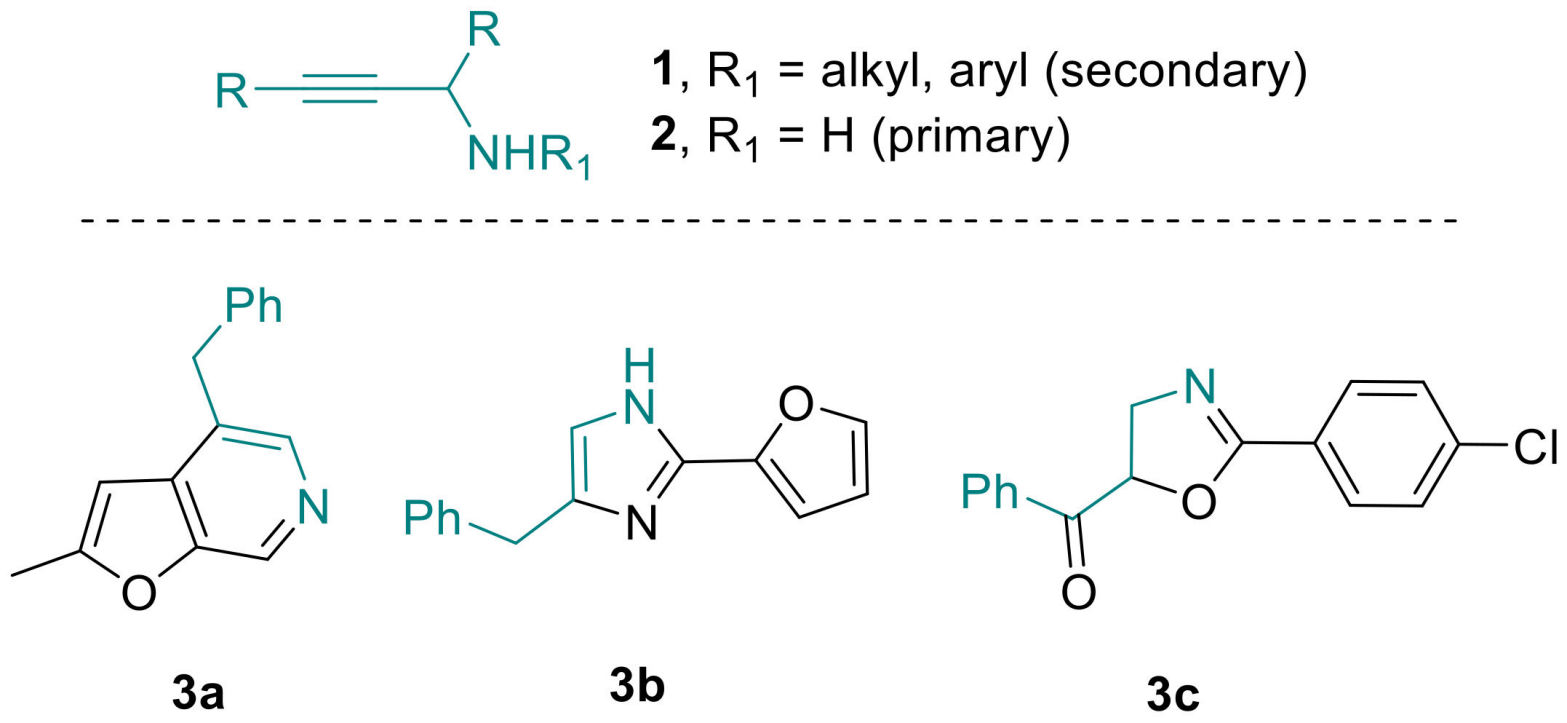
Propargylic amines and derived heterocycles.

As modern drug development programs continue to rely on suitably functionalized molecular building blocks, efforts towards their effective preparation remain a challenge at the forefront of modern synthetic chemistry. Oftentimes however, such key building blocks may not be readily available or cannot be stored for long periods of time due to unavoidable degradation, making on-demand synthesis the only option for synthetic chemists. Propargylic amines [1] represent such a distinct class of important building blocks that are invaluable for accessing various nitrogen-containing entities with widespread applications in the synthesis of bioactive structures. As a consequence, propargylic amines have recently featured prominently as key components in contemporary syntheses of pyridines [2], imidazoles [3] and oxazolines [4] (Scheme 1). 

**Scheme 2:**
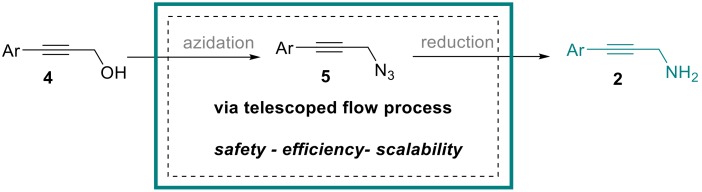
Telescoped flow approach to propargylic amines **2**.

Amongst the synthetic methods available for preparing secondary propargylic amines **1**, the classical metal-catalyzed three-component reaction between amines, alkynes and aldehydes stands out as the most widely utilized protocol [5,6,7,8,9,10]. Further options include the amination of allenes [11], the decarboxylative coupling of amines with alkyne carboxylic acids in the presence of formaldehyde [12], the copper-catalyzed coupling of aryl boronic acids with ammonia and propargylic halides [13] or the rhenium-catalyzed direct substitution of activated propargylic alcohols [14]. In contrast, the synthesis of primary propargylic amines **2** typically requires a stepwise and therefore more cumbersome approach that cannot be performed in a single-pot fashion. Therefore, the stepwise conversion of propargylic alcohols **4** via the activation and subsequent displacement of the hydroxy group with a suitable nitrogen nucleophile is typically required. Commonly, azide is the preferred nucleophile that subsequently must be converted into the desired amine functionality by suitable reduction processes (Scheme 2).

To support an ongoing synthesis program, we required a robust and scalable route towards a variety of different propargylic amines **2** bearing different aryl-substituents. In view of potential safety concerns that might manifest during reaction scale-up, we opted to develop a continuous flow protocol that would not only enable and streamline the assembly and delivery of these entities, but also mitigate any safety concerns associated with the introduction and subsequent reduction of the azide functionality. The latter is commonly accomplished under Staudinger reduction conditions releasing stoichiometric amounts of nitrogen gas in the process. Thus, we aimed at developing a telescoped process that would allow performing these two steps in a continuous flow reactor [15,16,17,18,19,20,21,22] to conveniently and safely [23] deliver a small selection of these propargylic amines on multi-gram scale.

## 2. Results

**Scheme 3:**
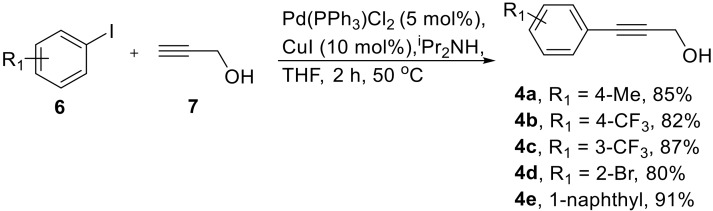
Sonogashira reaction yielding propargylic alcohols **4**.

As outlined in Scheme 2, propargylic alcohols were identified as suitable starting materials for the telescoped flow synthesis of the corresponding amine structures. The synthesis of a small selection of propargylic alcohols **4a–e** was readily accomplished in batch mode using typical Sonogashira coupling conditions between aryl iodides **6** and propargyl alcohol (**7**) giving gram quantities of the desired entities (Scheme 3). As no difference was noted between performing this reaction at small (1 mmol) or larger scale (20 mmol), no further optimization was required.

**Scheme 4:**
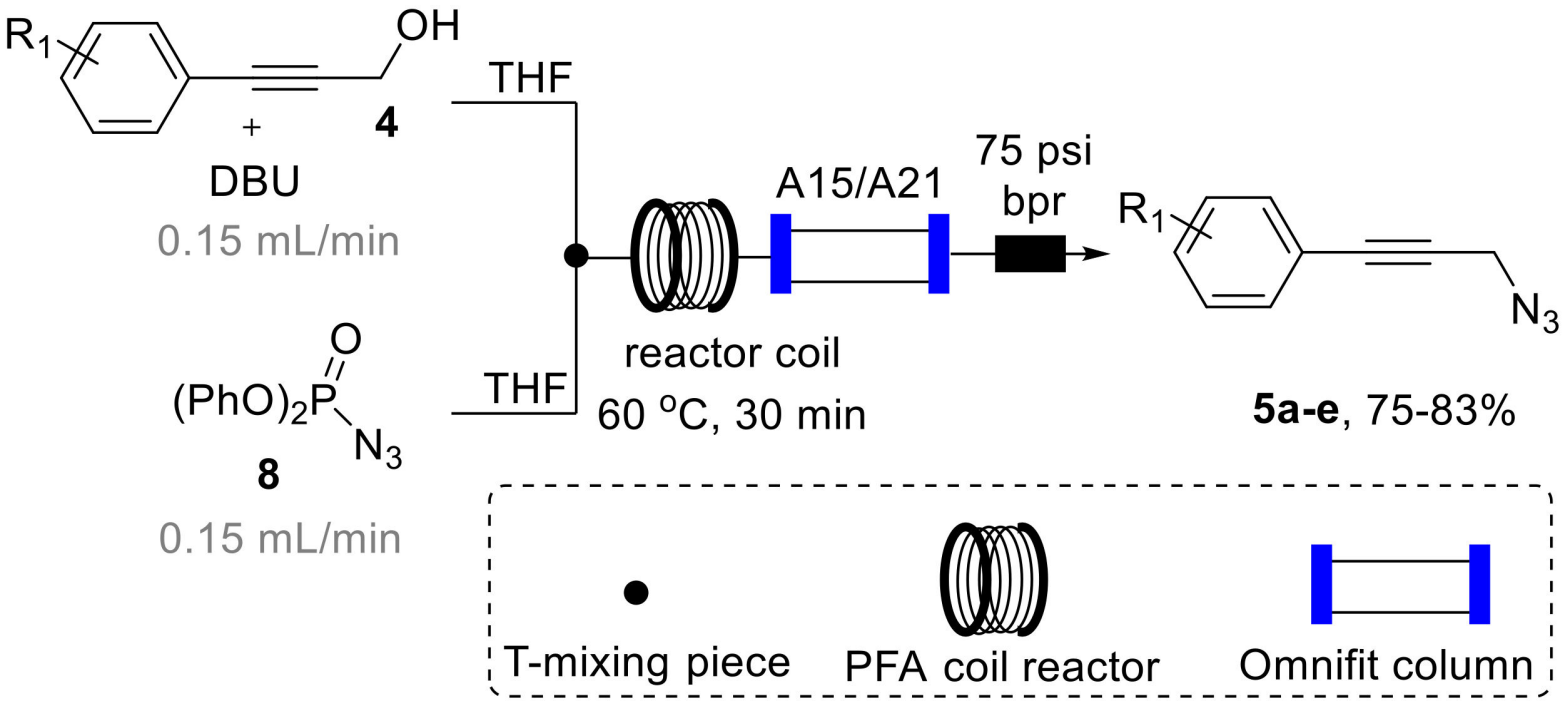
Azidation of **4** using DPPA (**8**) in continuous flow mode.

Having established a rapid entry into the desired substrates **4**, we turned our attention to their conversion into the propargylic amine targets in flow mode. As mentioned earlier, this was driven by the desire to streamline the synthesis effort avoiding time consuming isolation and purification stages for the potentially hazardous azide intermediate **5** as well as the release of nitrogen gas during the Staudinger reduction step, that could lead to a dangerous run-away process. To accomplish this, we opted to utilize diphenylphosphoryl azide (**8**, DPPA) as a readily available and bench stable azide donor that we [24] and others [25,26] had exploited previously in flow-based azide transformations. This rendered the opportunity to subsequently treat the resulting propargylic azide with triphenylphosphine in a suitable solvent system to affect the formation of the desired propargylic amines via a telescoped Staudinger reduction [27] sequence (Scheme 4).

As depicted in Scheme 4, we designed a flow process utilizing a Vapourtec R-series system that combines a solution of DPPA (**8**, THF, 2 M, 2 equiv., stream A) via a T-piece with a second stream containing the substrate (**4**, THF, 1 M, 1 equiv.) and DBU (1.3 equiv.) as a base that we had found to efficiently promote this transformation. The resulting mixture was then directed into a heated flow coil reactor (10 mL, PFA, 1/16′ i.d.) and collected after passing a back-pressure regulator (75 psi).

During an initial optimization study ^1^H-NMR analysis of the resulting reaction mixture revealed that elevated temperature (60 °C) in combination with residence times of 30 min were optimal to reach full conversion of **4** to the desired azide products **5** that are isolable entities. It was furthermore found that using an excess of DPPA (2 equiv.) was required to reliably achieve this transformation in a short period of time. Using these conditions allowed to generate the desired azide products (**5a–c**) in good isolated yields also permitting their full spectroscopic characterization (see Supplementary Materials for full details). Furthermore, on small scale (1 mmol) this flow process was successfully coupled with in-line scavenging of by-products and spent reagents by placing a mixed bed of sulfonic acid resin (Amberlyst A15) and an immobilized tertiary amine base (Amberlyst A21) in an Omnifit glass column (15 cm length, 1 cm i.d., containing ~3 equiv. of each species) to yield a mixture of the desired azide product and residual DPPA that could either be purified using column chromatography or directed into the subsequent Staudinger reduction process (vide infra) [28].

Subsequent batch test reactions for the desired Staudinger reduction of intermediates **5** had indicated that the transient iminophosphorane species **9** along with nitrogen gas is readily formed upon treatment of the azide intermediate with triphenylphosphine (**10**, 1.3 equiv. in THF). Furthermore, the hydrolysis of the iminophosphorane turned out to be a facile process yielding the desired propargylic amines upon addition of water (10% by volume) to this reaction mixture. 

**Scheme 5:**
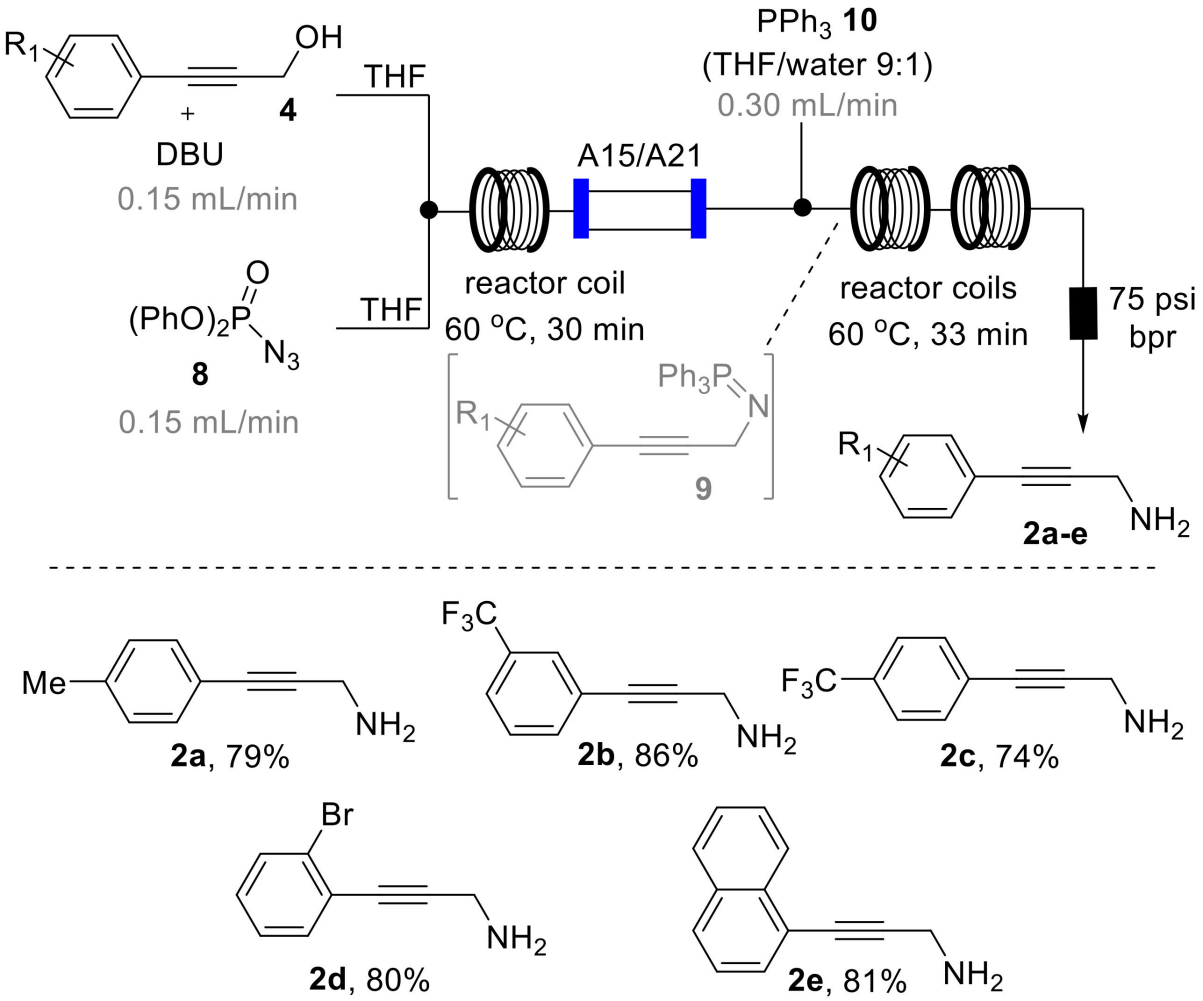
Telescoped azide formation and Staudinger reduction sequence in flow mode.

To translate these preliminary results into a telescoped flow process, we expanded on the original set-up by mixing the azide stream with a solution of triphenylphosphine (90:10 THF/water, 2.0 equiv., Scheme 5). To match the effective concentration of the azide intermediate **5** in the initial reaction stream and mitigate any dispersion-related problems a slight excess of the triphenylphosphine reagent (1.5 equiv.) was used [29]. The resulting reaction mixture was directed through two additional flow coils (PFA, 10 mL volume each) maintained at 60 °C (residence time: 33 min) before passing a back-pressure regulator (75 psi) and collection in a flask. Due to the continuous process and the related generation and removal of nitrogen gas as a by-product, no build-up of pressure was noted as this gaseous by-product was steadily removed in the course of the reaction. This highlights how flow processing circumvents the need for additional safety and process control measures and allows for directly scaling this sequence without recourse for further adaptations. At this stage the desired amine products **2a**–**e** were isolated after extractive acid-base work-up in good yields and excellent purity as listed in Scheme 5.

To further expand on this telescoped process and in view of effectively separating the desired amine product from residual DPPA, we opted to incorporate the acid-base extraction process within the flow sequence. This also addresses safety concerns regarding the co-isolation of larger quantities of unreacted DPPA when transitioning from small (1 mmol) to larger scale (5 to >10 mmol) operations. To this end an additional HPLC pump was used to deliver a stream of aqueous acid (HCl, 1 M, 0.6 mL/min) that was mixed via a T-piece with the crude mixture from the azidation/Staudinger reduction sequence.

**Scheme 6:**
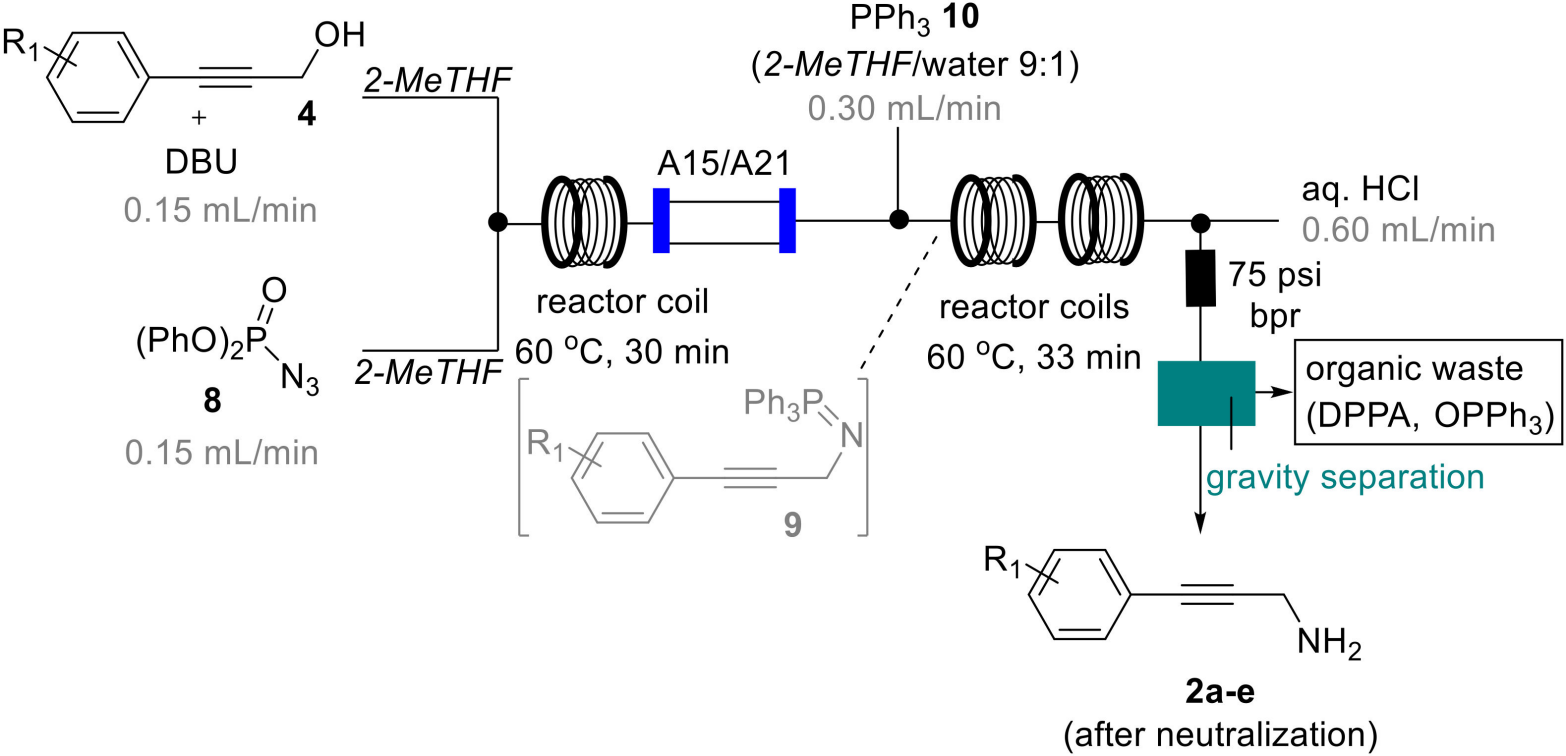
Final telescoped flow process using 2-MeTHF.

After collection of the resulting biphasic mixture in a separating funnel, the desired propargylic amine products were again obtained in pure form upon neutralization and back-extraction into either DCM or EtOAc. Although the mixing in a conventional T-piece aided in emulsifying and consequently protonating the propargylic amine species, it was found that THF as reaction solvent led to slow phase separation and thus potential loss of product (around 10–15%). A quick reevaluation of suitable solvent alternatives revealed that 2-methyltetrahydrofuran (2-MeTHF) could effectively replace THF as it resulted in faster phase separation. Additionally, this solvent swap would be attractive as 2-MeTHF is reportedly more stable towards acids, whilst being both bioderived and biodegradable [30,31,32]. The final flow process (Scheme 6) thus used 2-MeTHF as sole organic solvent and allowed the effective generation of the desired propargylic amines as well as in-line separation from by-products and excess azide donor (DPPA).

**Figure 1:**
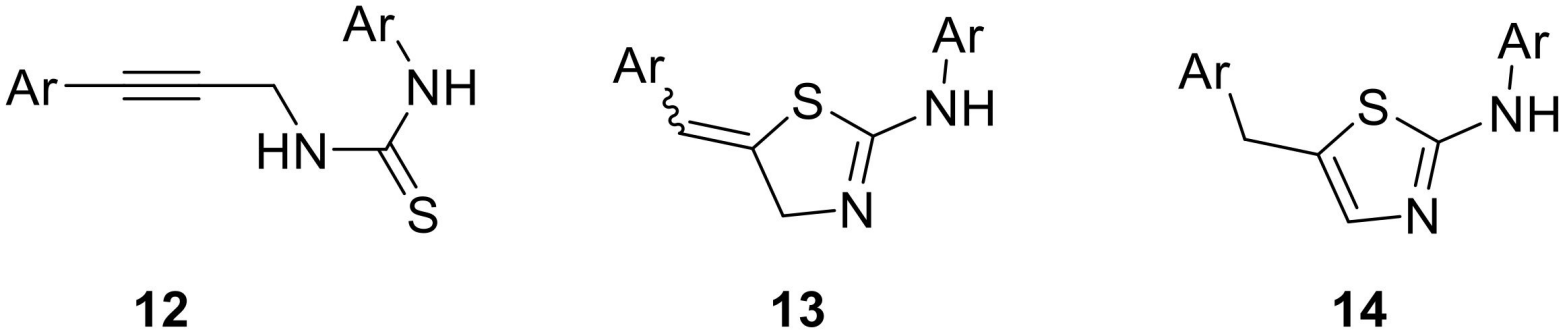
Structure of thiazolines **13** and thiazoles **14** derived from propargylic thioureas **12**.

To exploit our effective route into different aryl-propargyl amines and demonstrate the value of the flow process developed to generate these materials on-demand, we studied their reaction with different isothiocyanates **11**. Such a process was expected to yield valuable heterocyclic derivatives via intermediate thiourea adducts **12** that would cyclize in an acid-catalyzed reaction cascade. This sequence is interesting as it allows direct access to either thiazoline or thiazole derivatives **13** or **14** as reported in the literature [33,34,35,36,37]. We were specifically interested in selectively generating thiazoline species as these provide an exocyclic alkene that can serve as a handle for accessing highly functionalized derivatives (Figure 1).

**Scheme 7:**
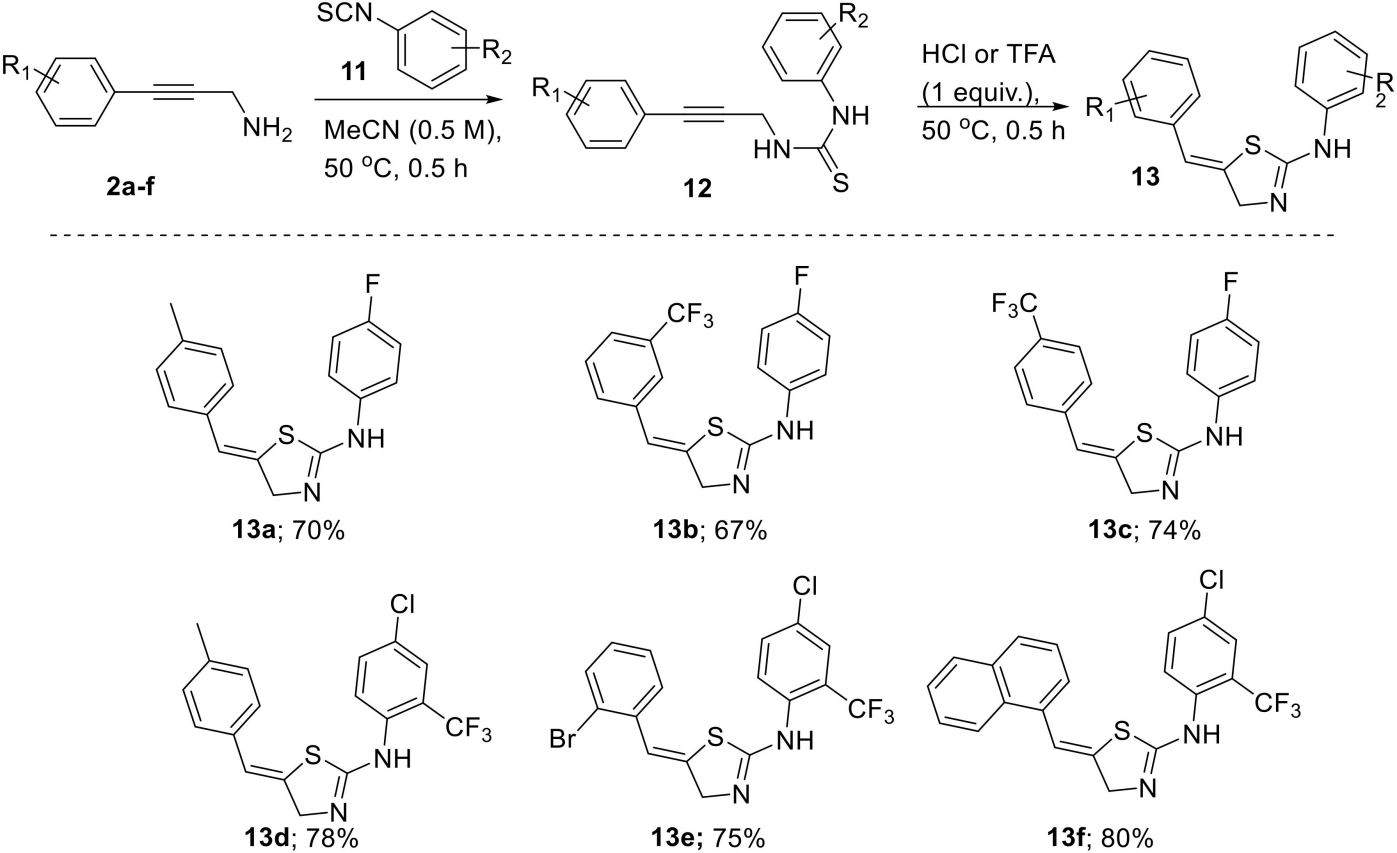
Cyclization cascade towards thiazoline products **13**.

To this end we treated a solution of the respective propargylic amine **2** (0.5 M, MeCN) with stoichiometric amounts of suitable aryl isothiocyanates (**11**). The expected thiourea intermediate **12** formed within 30 min and upon addition of acid (HCl or TFA, 1.0 equiv.) smoothly converted into the heterocyclic target structures within a short time (Scheme 7).

**Figure 2:**
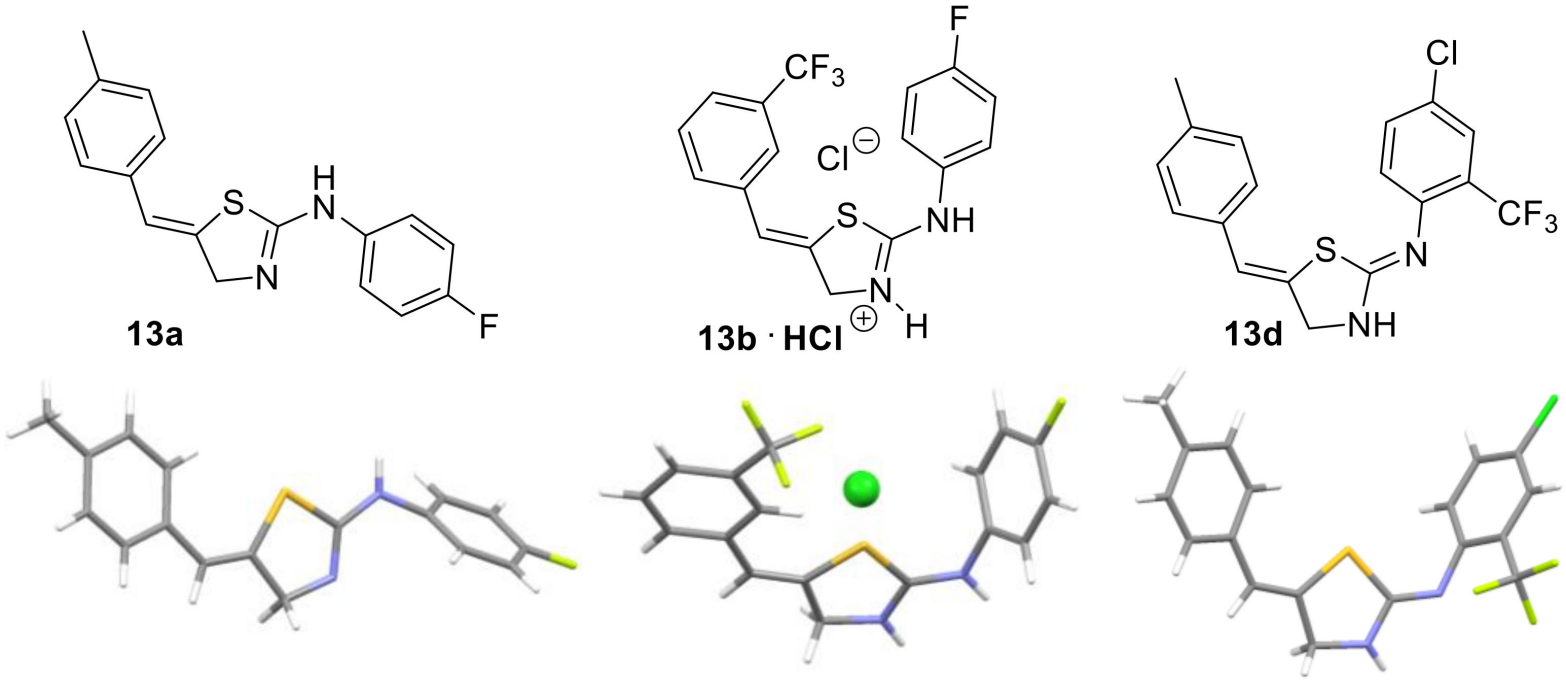
X-ray crystallographic study on selected thiazolines.

Encouraged by these results we opted to verify the provisional structural assignment of our products as being thiazolines bearing an exocyclic alkene. This was warranted as conventional spectroscopic techniques were not allowing for an unambiguous assignment and literature precedent was not clear. We thus used single crystal diffraction experiments to prove that the expected thiazolines had indeed formed. Importantly, this allowed to demonstrate that thiazoline products were obtained in all cases independent whether a base was employed during the work-up as summarized in Figure 2. 

These results furthermore confirm the *Z*-configuration of the alkene and demonstrate that the thiazoline tautomer is favored over the aromatic thiazole structure, highlighting the relevance of an extended π-conjugated system over the aromaticity of the alternative thiazole heterocycle.

## 3. Materials and Methods

### 3.1. General Information

Unless otherwise stated, all solvents were purchased from Fisher Scientific and used without further purification. Substrates and reagents were purchased from Fluorochem or Sigma Aldrich and used as received.

^1^H-NMR spectra were recorded on 300 or 400 MHz instruments and are reported relative to residual solvent: CHCl_3_ (δ 7.26 ppm). ^13^C-NMR spectra were recorded on the same instruments (100 MHz) and are reported relative to CHCl_3_ (δ 77.16 ppm). Data for ^1^H-NMR are reported as follows: chemical shift (δ/ppm) (integration, multiplicity, coupling constant (Hz)). Multiplicities are reported as follows: s = singlet, d = doublet, t = triplet, q = quartet, p = pentet, m = multiplet, br. s = broad singlet, app = apparent. Data for ^13^C-NMR are reported in terms of chemical shift (δ/ppm) and multiplicity (C, CH, CH_2_ or CH_3_). DEPT-135, COSY, HSQC, HMBC and NOESY experiments were used in the structural assignment. IR spectra were obtained by use of a Platinum spectrometer (neat, ATR sampling, Bruker, Billerica, MA, USA) with the intensities of the characteristic signals and are reported as weak (w, <20% of tallest signal), medium (m, 21–70% of tallest signal) or strong (s, >71% of tallest signal). High-resolution mass spectrometry was performed using the indicated techniques on a Micromass LCT orthogonal time-of-flight mass spectrometer (Micromass, Manchester, UK with leucine-enkephalin (Tyr-Gly-Phe-Leu) as an internal lock mass. Continuous flow experiments were performed on a Vapourtec E-series system (Vapourtec, Bury Saint Edmunds, UK) in conjunction with Omnifit glass columns.

### 3.2. General Procedure for the Synthesis of Propargyl Alcohols ***4a**–**e***

To a solution of aryl iodide (10 mmol, 1.0 equiv.) in THF (10 mL, 1.0 M) was added 2-propyn-1-ol (1.24 mL, 1.2 equiv.), diisopropylamine (1.68 mL, 1.2 equiv.), PdCl_2_(PPh_3_)_2_ (0.3 mmol, 3 mol%) and CuI (0.5 mmol, 5 mol%). The resulting solution changed colour from yellow to orange and finally brown in two minutes. The reaction mixture was heated at 50 °C and stirred until complete consumption of the aryl iodide was observed (tlc, 2 h). After filtering the crude material over a pad of silica (5 g) and evaporation of the solvent the crude material (^1^H-NMR) was purified by silica column chromatography (eluent 10% EtOAc in pentanes, Rf = 0.41). After removal of the volatiles, the desired hydroxyl product was typically isolated as yellow oil with a purity > 98% (by ^1^H-NMR) and was used directly in the next step. Copies of NMR spectra are provided in the electronic Supplementary Materials.

### 3.3. General Procedure for the Synthesis of Propargyl Azides ***5a**–**e***

A flow set-up was constructed in which a first stream containing DPPA (2 M, THF, 2 equiv.) was mixed with a stream containing the substrate (**4a**–**e**, 1 M, THF, 1 equiv.) and DBU (1.3 equiv.). Each stream was pumped at a flow rate of 0.15 mL/min and blended in a T-piece. The resulting mixture was passed into a tubular flow coil (10 mL, PFA, 60 °C, ~30 min residence time) to affect the azidation reaction. The exiting stream was directed through an Omnifit glass column (10 cm x 1.0 cm i.d., ambient temperature) containing a mixture of washed scavenger resins (A21 and A15, ~1:1 ratio, 3 equiv. each) to remove acidic and basic by-products. After passing a back-pressure regulator (75 psi) the product was collected in a flask. Purification was achieved by silica column chromatography (2–5% EtOAc/hexanes) giving the desired products typically as oils.

### 3.4. General Procedure for the Synthesis of Propargyl Amines ***2a**–**e***

To realise the Staudinger reduction step in a telescoped flow process, the purified stream of the intermediate azide solution (see above) was combined in a T-piece with a stream of triphenylphosphine (2 equiv.) in aqueous THF (THF/water, 9:1) at equal flow rates of 0.3 mL/min. The resulting mixture then entered two consecutive flow coils (PFA, 10 mL each, 60 °C, residence time ~33 min) before passing a back-pressure regulator (75 psi) and collection in a flask. After evaporation of the volatiles, the crude mixture was partitioned between DCM (2 × 25 mL) and aqueous HCl (1 M). After discarding the organic phase, the pH of the aqueous layer was adjusted to 8–9 and the mixture was extracted with DCM (2 × 25 mL). The combined organic extracts were dried over anhydrous sodium sulfate, filtered and evaporated in vacuo giving the desired products either as oils or amorphous solids.

### 3.5. General Procedure for the Synthesis of Thiazolines ***13a**–**e***

To a solution of the amine **2a**–**e** (1 equiv.) in MeCN (0.5 M) was added a stoichiometric amount of aryl isothiocyanate. The resulting mixture was stirred at 50 °C for 30 min before addition of acid (TFA or HCl, 1 equiv.). Once complete consumption of substrates to a new product was observed by tlc (0.5-1 h), the pH was adjusted to 7 by addition of Na_2_CO_3_ (sat. aqueous). After evaporation of volatiles under reduced pressure, the crude material was triturated from mixtures of MeCN and water (4:1). Alternatively, silica column chromatography (5–20% EtOAc/hexanes was used to obtain pure thiazoline products as amorphous solids that can be recrystallized to grow single crystals (MeOH/DCM).

*3-(p-Tolyl)prop-2-yn-1-ol* (**4a**): Yield: 85% (1.2 g; 8.3 mmol). Appearance: yellow oil. ^1^H-NMR (400 MHz, CDCl_3_) δ 7.33 (d, *J* = 8.1 Hz, 2H), 7.12 (d, *J* = 8.1 Hz, 2H), 4.48 (d, *J* = 6.1 Hz, 2H), 2.35 (s, 3H), 1.72 (t, *J* = 6.1 Hz, 1H). ^13^C-NMR (100 MHz, CDCl_3_) δ 138.7 (C), 131.6 (2CH), 129.1 (2CH), 119.4 (C), 86.5 (C), 85.7 (C), 51.7 (CH_2_), 21.5 (CH_3_). IR (neat, cm^−1^) 3296 (m), 2919 (m), 2863 (m), 2237 (s), 1651 (w), 1607 (w), 1562 (w), 1509 (s), 1407 (m), 1379 (m), 1260 (m), 1026 (s), 816 (s). Rf = 0.41 (10% EtOAc in pentanes). HR-MS (TOF-ES+) calculated for C_10_H_10_O 146.0732, found 146.0716 (Δ = −4.1 ppm).

*3-(4-(Trifluoromethyl)phenyl)prop-2-yn-1-ol* (**4b**): Yield: 82% (1.4 g; 7.0 mmol). Appearance: yellow oil. ^1^H-NMR (400 MHz, CDCl_3_) δ 7.55 (d, *J* = 8.0 Hz, 2H), 7.51 (d, *J* = 8.0 Hz, 2H), 4.51 (s, 2H), 2.16 (s, H). ^13^C-NMR (100 MHz, CDCl_3_) δ 131.9 (2CH), 130.1 (q, *J* = 30.5 Hz, C), 126.3 (br s, C), 125.5 (q, *J* = 4.0 Hz, 2CH), 123.8 (q, *J* = 273 Hz, CF_3_), 89.6 (C), 84.3 (C), 51.4 (CH_2_). ^19^F-NMR (376 MHz, CDCl_3_) δ −62.9. Rf = 0.44 (10% EtOAc in pentanes). IR (neat, cm^−1^) 3256 (m), 2920 (w), 2863 (w), 2242 (w), 1929 (w),1803 (w), 1685 (w), 1614 (m), 1567 (w), 1481 (w),1403 (w), 1316 (s), 1170 (s), 1119 (s), 1104 (s), 1015 (s), 952 (m), 841 (s), 710 (m), 598 (m). HR-MS (TOF-ES+) calculated for C_10_H_7_F_3_O 200.0449, found 200.0445 (Δ = −2.0 ppm).

*3-(3-(Trifluoromethyl)phenyl)prop-2-yn-1-ol* (**4c**): Yield: 87% (1.6 g; 8.2 mmol). Appearance: yellow oil. ^1^H-NMR (400 MHz, CDCl_3_) δ 7.68 (s, 1H), 7.57 (t, *J* = 12.6 Hz, 2H), 7.41 (t, *J* = 9.0 Hz, 1H), 4.50 (d, *J* = 3.0 Hz, 2H), 2.25 (s, 1H).^13^C-NMR (100 MHz, CDCl_3_) δ 134.7 (m, CH), 130.9 (q, *J* = 32.5 Hz, CH), 128.8 (CH), 128.4 (q, *J* = 4.0 Hz, C), 125.0 (q, *J* = 4.0 Hz, CH), 123.6 (q, *J* = 274.1 Hz, CF_3_) 123.5 (C), 88.8 (C), 84.1 (C), 51.4 (CH_2_). ^19^F-NMR (376 MHz, CDCl_3_) δ −63.1. Rf = 0.43 (10% EtOAc in pentanes). IR (neat, cm^−1^) 3297 (m), 2917 (s), 2868 (s), 2112 (s), 1611 (w), 1588 (w), 1486 (m), 1433 (m), 1331 (s), 1237 (s), 1125 (s), 1029 (m), 973 (m), 902 (m), 802 (s), 696 (s). HR-MS (TOF-ES+) calculated for C_10_H_7_F_3_O 200.0449, found 200.0453 (Δ = 2.0 ppm).

*3-(2-Bromophenyl)prop-2-yn-1-ol* (**4d**): Yield: 80% (0.84 g, 4.0 mmol). Appearance: yellow oil. ^1^H-NMR (400 MHz, CDCl_3_) δ 7.57 (dd, *J* = 7.9, 1.4 Hz, 1H), 7.47 (dd, *J* = 7.8, 1.7 Hz, 1H), 7.26 (td, *J* = 7.7, 1.3 Hz, 1H), 7.17 (td, *J* = 7.7, 1.8 Hz, 1H), 4.55 (d, *J* = 5.9 Hz, 2H), 1.78 (t, *J* = 6.1 Hz, 1H). ^13^C-NMR (100 MHz, CDCl_3_) δ 133.5 (CH), 132.4 (CH), 129.7 (CH), 127.0 (CH), 125.4 (C), 124.7 (C), 91.8 (C), 84.2 (C), 51.7 (CH2). IR (neat, cm^−1^) 3303 (broad), 2913 (w), 1587 (w), 1468 (s), 1433 (m), 1052 (m), 1023 (s), 953 (m), 749 (s), 653 (m), 574 (m).

*3-(Naphthalen-1-yl)prop-2-yn-1-ol* (**4e**): Yield: 91% (0.83 g, 4.6 mmol). Appearance: yellow oil. ^1^H-NMR (400 MHz, CDCl_3_) δ 8.36 (dd, *J* = 8.1, 1.1 Hz, 1H), 7.87–7.80 (m, 2H), 7.71 – 7.67 (m, 1H), 7.58 (ddd, *J* = 8.3, 6.8, 1.5 Hz, 1H), 7.55–7.49 (m, 1H), 7.43–7.38 (m, 1H), 4.67 (s, 2H), 2.45 (s, 1H). ^13^C-NMR (100 MHz, CDCl_3_) δ 133.3 (C), 133.1 (C), 130.7 (CH), 129.0 (CH), 128.3 (CH), 126.8 (CH), 126.4 (CH), 126.1 (CH), 125.2 (CH), 120.2 (C), 92.2 (C), 83.8 (C), 51.8 (CH_2_). IR (neat, cm^−1^) 3040 (w), 2933 (w), 2170 (w), 2121 (m), 1643 (s), 1589 (s), 1487 (s), 1249 (s), 1205 (s), 1161 (m), 1090 (s), 961 (m), 896 (s), 771 (s), 680 (s), 528 (s). HR-MS (TOF-ES+) calculated for C_13_H_9_ (M-CH_2_OH) 165.0704, found 165.0706 (Δ = 1.1 ppm).

*1-(3-Azidoprop-1-yn-1-yl)-4-methylbenzene* (**5a**): Yield: 75% (0.8 g; 4.7 mmol). Appearance: yellow oil. ^1^H-NMR (400 MHz, CDCl_3_) δ 7.36 (d, *J* = 8.2 Hz, 2H), 7.13 (d, *J* = 8.8 Hz, 2H), 4.13 (s, 2H), 2.36 (s, 3H). ^13^C-NMR (100 MHz, CDCl_3_) δ 139.0 (C), 131.8 (2CH), 129.1 (2CH), 118.9 (C), 87.6 (C), 80.3 (C), 40.6 (CH_2_), 21.5 (CH_3_). Rf = 0.80 (5% EtOAc in pentanes). IR (neat, cm^−1^) 3029 (w), 2920 (w), 2860 (w), 2217 (w), 2116 (s), 1607 (w), 1509 (s), 1442 (m), 1338 (m), 1267 (m), 1238 (s), 865 (m), 867 (s), 732 (m). HR-MS (TOF-ES+) calculated for C_10_H_9_N (M-N_2_) 143.0735, found 143.0739 (Δ = 2.8 ppm).

*1-(3-Azidoprop-1-yn-1-yl)-3-(trifluoromethyl)benzene* (**5b**): Yield: 83% (0.8 g; 3.6 mmol). Appearance: yellow oil. ^1^H-NMR (300 MHz, CDCl_3_) δ 7.73 (s, H), 7.63 (t, *J* = 10.9 Hz, 2H), 7.47 (t, *J* = 7.8 Hz, H), 4.17 (s, 2H). ^13^C-NMR (75 MHz, CDCl_3_) δ 135.0 (CH), 131.0 (q, *J* = 34 Hz, C), 128.9 (CH), 128.6 (q, *J* = 4 Hz, CH), 125.4 (q, *J* = 4 Hz, CH), 123.6 (q, *J* = 272 Hz, CF_3_),122.9 (C), 85.4 (C), 82.8 (C), 40.4 (CH_2_). ^19^F-NMR (282 MHz, CDCl_3_) δ −63.0. Rf = 0.78 (5% EtOAc in pentanes). IR (neat, cm^−1^) 3080 (w), 2940 (w), 2105 (s), 1635 (s), 1590 (s), 1486 (m), 1434 (m), 1331 (s), 1237 (m), 1167 (s), 1127 (s), 1095 (m), 1747 (m), 1005 (m), 903 (m), 803 (s), 695 (s), 656 (m). HR-MS (TOF-ES+) calculated for C_10_H_6_F_3_N (M-N_2_)197.0452, found 197.0450 (Δ = −1.0 ppm).

*1-(3-Azidoprop-1-yn-1-yl)-4-(trifluoromethyl)benzene* (**5c**): Yield: 77% (0.7 g; 3.1 mmol). Appearance: yellow oil. ^1^H-NMR (400 MHz, CDCl_3_) δ 7.55-7.60 (m, 4H), 4.16 (s, 2H). ^13^C-NMR (100MHz, CDCl_3_) δ 132.1 (2CH), 130.6 (q, *J* = 33 Hz, C), 125.7 (br s, C) 125.3 (q, *J* = 4 Hz, 2CH), 125.8 (q, *J* = 272 Hz, CF_3_), 85.9 (C), 83.6 (C), 40.4 (CH_2_). ^19^F-NMR (376 MHz, CDCl_3_) δ −63.0. Rf = 0.78 (5% EtOAc in pentanes). IR (neat, cm^−1^) 2916 (w), 2115 (s), 1922 (s), 1570 (m), 1406 (m), 1321 (s), 1269 (m), 1241 (m), 1166 (m), 1126 (s), 1106 (m), 1068 (s), 1018 (m), 842 (s), 688 (s), 554 (m). HR-MS (TOF-ES+) calculated for C_10_H_6_F_3_N (M-N_2_) 197.0452, found 197.0458 (Δ = 3.0 ppm).

*1-(3-Azidoprop-1-yn-1-yl)-2-bromobenzene* (**5d**): Yield: 82% (0.7 g; 2.9 mmol). Appearance: yellow oil. ^1^H-NMR (400 MHz, CDCl_3_) δ 7.59 (dd, *J* = 7.9, 1.3 Hz, 1H), 7.50 (dd, *J* = 7.6, 1.8 Hz, 1H), 7.30–7.25 (m, 1H), 7.20 (td, *J* = 7.7, 1.7 Hz, 1H), 4.19 (s, 2H). ^13^C-NMR (100 MHz, CDCl_3_) δ 133.8 (CH), 132.5 (CH), 130.0 (CH), 127.0 (CH), 125.4 (C), 124.2 (C), 85.8 (C), 85.6 (C), 40.6 (CH_2_). IR (neat, cm^−1^) 2910 (w), 2119 (s), 2100 (s), 1469 (s), 1434 (m), 1335 (m), 1246 (m), 1233 (m), 1052 (m), 1026 (m), 988 (m), 865 (m), 750 (s), 688 (m), 650 (m), 552 (m), 512 (m), 445 (m). HR-MS (TOF-ES+) calculated for C_9_H_6_Br (M-N_3_) 192.9653, found 192.9657 (Δ = 2.1 ppm).

*1-(3-Azidoprop-1-yn-1-yl)naphthalene* (**5e**): Yield: 79% (0.67 g; 3.2 mmol). Appearance: pale yellow oil. ^1^H-NMR (400 MHz, CDCl_3_) δ 8.33 (d, *J* = 8.1 Hz, 1H), 7.89–7.85 (m, 2H), 7.73 (dd, *J* = 7.0, 1.2 Hz, 1H), 7.60 (ddd, *J* = 8.3, 6.9, 1.4 Hz, 1H), 7.56–7.51 (m, 1H), 7.44 (dd, *J* = 8.4, 7.2 Hz, 1H), 4.31 (s, 2H). ^13^C-NMR (100 MHz, CDCl_3_) δ 133.1 (C), 133.1 (C), 131.1 (CH), 129.4 (CH), 128.3 (CH), 127.0 (CH), 126.5 (CH), 125.9 (CH), 125.1 (CH), 119.6 (C), 85.8 (C), 85.4 (C), 40.9 (CH2). IR (neat, cm^−1^) 3058 (w), 2119 (s), 2094 (s), 1586 (w), 1507 (w), 1395 (m), 1335 (m), 1247 (s), 865 (m), 797 (s), 770 (s).

*3-(p-Tolyl)prop-2-yn-1-amine* (**2a**): Yield: 79% (0.45 g; 3.2 mmol). Appearance: yellow solid. ^1^H-NMR (400 MHz, CDCl_3_) δ 7.29 (d, *J* = 8.1 Hz, 2H), 7.10 (d, *J* = 8.1 Hz, 2H), 3.63 (s, 2H), 2.33 (s, 3H). ^13^C-NMR (100 MHz, CDCl_3_) δ 138.1 (C), 131.4 (2CH), 129.0 (2CH), 120.1 (C), 89.5 (C), 82.5 (C), 32.2 (CH_2_), 21.4 (CH_3_). IR (neat, cm^−1^) 3347 (w), 3265 (m), 3027 (w), 2916 (w), 2247 (w), 2131 (w), 1906 (s), 1607 (s), 1555 (m), 1433 (s), 1330 (s), 1306 (s), 969 (m), 813 (s), 544 (m), 495 (m). HR-MS (TOF-ES+) calculated for C_10_H_12_N (M + H) 146.0970, found 146.0969 (Δ = −0.5 ppm).

*3-(3-(Trifluoromethyl)phenyl)prop-2-yn-1-amine* (**2b**): Yield: 86% (0.5 g; 2.6 mmol). Appearance: yellow oil. ^1^H-NMR (400 MHz, CDCl_3_) δ 7.67 (s, 1H), 7.55 (m, 2H), 7.41 (t, *J* = 7.9 Hz, 1H), 3.66 (s, 2H). ^13^C-NMR (100 MHz, CDCl_3_) δ 134.6 (m, CH), 130.9 (m, C), 128.8 (CH), 128.4 (q, *J* = 3 Hz, CH), 124.6 (q, *J* = 4 Hz, CH), 124.2 (C), 123.7 (q, *J* = 274 Hz, CF_3_), 91.8 (C), 81.0 (C), 32.1 (CH_2_). ^19^F-NMR (376 MHz, CDCl_3_) δ −63.0. IR (neat, cm^−1^) 2919 (w), 2118 (w), 1587 (w), 1486 (m), 1328 (s), 1236 (m), 1163 (m), 1123 (s), 1073 (m), 1001 (w), 902 (m), 696 (m), 658 (s). HR-MS (TOF-ES+) calculated for C_10_H_9_NF_3_ (M + H) 200.0687, found 200.0683 (Δ = −2.0 ppm).

*3-(4-(Trifluoromethyl)phenyl)prop-2-yn-1-amine* (**2c**): Yield: 74% (0.44 g; 3.0 mmol). Appearance: yellow oil. ^1^H-NMR (400 MHz, CDCl_3_) δ 7.53 (d, *J* = 8.2 Hz, 2H), 7.48 (d, *J* = 8.2 Hz, 2H), 3.66 (s, 2H), 1.47 (br s, 2H). ^13^C-NMR (100 MHz, CDCl_3_) δ 131.8 (2CH), 129.7 (q, *J* = 33 Hz, C), 127.0 (br, C), 125.2 (q, *J* = 4 Hz, 2CH), 123.9 (q, *J* = 272 Hz, CF_3_), 92.9 (C), 81.1 (C), 32.0 (CH_2_). ^19^F-NMR (376 MHz, CDCl_3_) δ −62.9. IR (neat, cm^−1^) 3285 (w), 2921 (w), 2851 (w), 2239 (w), 1614 (m), 1437 (s), 1321 (s), 1261 (w), 1164 (s), 1122 (s), 1005 (m), 1168 (s), 1017 (m), 967 (s), 598 (m), 577 (w). HR-MS (TOF-ES+) calculated for C_10_H_9_NF_3_ (M + H) 200.0687, found 200.0697 (Δ = 5.0 ppm).

*3-(2-Bromophenyl)prop-2-yn-1-amine* (**2d**): Yield: 80% (0.42 g; 2.0 mmol). Appearance: yellow oil. ^1^H-NMR (400 MHz, CDCl_3_) δ 7.56 (dd, *J* = 8.1, 1.3 Hz, 1H), 7.43 (dd, *J* = 7.7, 1.7 Hz, 1H), 7.23 (td, *J* = 7.7, 1.6 Hz, 1H), 7.13 (td, *J* = 7.7, 1.7 Hz, 1H), 3.70 (s, 2H), 1.51 (s, 2H). ^13^C-NMR (100 MHz, CDCl_3_) δ 133.3 (CH), 132.3 (CH), 129.2 (CH), 127.0 (CH), 125.4 (C), 125.3 (C), 81.1 (C), 32.3 (CH_2_); 1 C not observed. IR (neat, cm^−1^) 3367 (w), 2914 (w), 1586 (w), 1468 (s), 1433 (m), 1330 (m), 1067 (m), 1048 (s), 1025 (m), 967 (m), 860 (m), 749 (s), 652 (m), 524 (m), 445 (m). HR-MS (TOF-ES+) calculated for C_9_H_9_NBr (M + H) 209.9918, found 209.9924 (Δ = 2.7 ppm).

*3-(Naphthalen-1-yl)prop-2-yn-1-amine* (**2e**): Yield: 81% (0.44 g; 2.4 mmol). Appearance: colourless oil. ^1^H-NMR (400 MHz, CDCl_3_) δ 8.33 (d, *J* = 8.2 Hz, 1H), 7.84 (dd, *J* = 8.0, 1.4 Hz, 1H), 7.81 (d, *J* = 8.2 Hz, 1H), 7.65 (d, *J* = 1.2 Hz, 0H), 7.56 (ddd, *J* = 8.3, 6.7, 1.5 Hz, 1H), 7.51 (ddd, *J* = 8.1, 6.8, 1.5 Hz, 1H), 7.44–7.39 (m, 1H), 3.81 (s, 2H), 1.56 (s, 2H). ^13^C-NMR (100 MHz, CDCl_3_) δ 133.3 (C), 133.1 (C), 130.2 (CH), 128.5 (CH), 128.2 (CH), 126.6 (CH), 126.3 (CH), 126.1 (CH), 125.2 (CH), 120.8 (C), 95.2 (C), 80.5 (C), 32.5 (CH2). IR (neat, cm^−1^) 3368 (w), 3056 (w), 2913 (w), 1584 (m), 1506 (w), 1395 (m), 1330 (m), 948 (m), 862 (m), 797 (s), 770 (s), 566 (m). HR-MS (TOF-ES+) calculated for C_13_H_12_N (M + H) 182.0970, found 182.0969 (Δ = −0.4 ppm).

*(Z)-N-(4-Fluorophenyl)-5-(4-methylbenzylidene)-4,5-dihydrothiazol-2-amine* (**13a**): Yield: 70% (0.42 g; 1.4 mmol). Appearance: yellow solid. ^1^H-NMR (400 MHz, d_4_-MeOD) δ 7.44–7.47 (m, 2H), 7.26–7.32 (m, 2H), 7.24 (d, *J* = 8.2 Hz, 2H), 7.19 (d, *J* = 8.2 Hz, 2H), 6.94 (t, *J* = 2 Hz, H), 4.95 (d, *J* = 2.4 Hz, 2H), 2.34 (s, 3H). ^13^C-NMR (100 MHz, d_4_-MeOD) δ 162.4 (d, *J* = 249 Hz, CF), 138.4 (C), 132.1 (C), 129.2 (2CH), 127.4 (2CH), 127.0 (d, *J* = 9 Hz, 2CH), 124.2 (C), 124.1 (CH), 116.8 (d, *J* = 24 Hz, 2CH), 56.0 (CH_2_), 19.9 (CH_3_), 2 carbons not observed. ^19^F-NMR (376 MHz, d_4_-MeOD) δ −113.7. IR (neat, cm^−1^) 3117 (w), 3048 (m), 2855 (m), 2675 (s), 1605 (s), 1555 (m), 1507 (s), 1460 (m), 1352 (s), 845 (s), 790 (m), 650 (m), 514 (m). HR-MS (TOF-ES+) calculated for C_17_H_15_FN_2_S (M + H) 299.1018, found 299.1008 (Δ = −3.4 ppm).

*(Z)-N-(4-Fluorophenyl)-5-(3-(trifluoromethyl)benzylidene)-4,5-dihydrothiazol-2-amine* (**13b**): Yield: 67% (0.47 g; 1.3 mmol). Appearance: colourless solid. ^1^H-NMR (400 MHz, d_4_-MeOD) δ 7.58–7.67 (m, 4H), 7.46–7.49 (m, 2H), 7.29 (t, *J* = 9.1 Hz, 2H), 7.06 (t, *J* = 2.3 Hz, H), 5.02 (d, *J* = 2.4 Hz, 2H). ^13^C-NMR (100 MHz, d_4_-MeOD) δ 162.5 (d, *J* = 248 Hz, CF), 136.0 (C), 131.5 (C), 130.9 (q, *J* = 32 Hz, C), 130.6 (CH), 129.6 (CH), 128.3 (C), 127.0 (d, *J* = 9 Hz, 2CH), 124.4 (q, *J* = 4 Hz, CH), 124.2 (q, *J* = 4 Hz, CH), 123.9 (CF_3_, q, *J* = 272 Hz), 122.4 (CH), 116.8 (d, *J* = 24 Hz, 2CH), 56.2 (CH_2_); 1 C not observed. ^19^F-NMR (376 MHz, d_4_-MeOD) δ −64.4 (3F), −113.6 (1F). IR (neat, cm^−1^) 3390 (w), 3190 (m), 2827 (m), 2650 (m), 1629 (s), 1510 (s), 1487 (s), 1170 (s), 909 (m), 878 (s), 797 (m), 758 (m), 645 (m), 520 (w). HR-MS (TOF-ES+) calculated for C_17_H_13_N_2_F_4_S (M + H) 357.0736, found 353.0737 (Δ = 0.4 ppm).

*(Z)-N-(4-Fluorophenyl)-5-(4-(trifluoromethyl)benzylidene)-4,5-dihydrothiazol-2-amine* (**13c**): Yield: 74% (0.52 g; 1.5 mmol). Appearance: colourless solid. 1H-NMR (400 MHz, d_4_-MeOD) δ 7.74 (d, *J* = 8.2 Hz, 2H), 7.52 (d, *J* = 8.2 Hz, 2H), 7.47–7.50 (m, 2H), 7.30 (t, *J* = 8.8 Hz, 2H), 7.07 (br s, H), 5.04 (d, *J* = 2.1 Hz, 2H). ^13^C-NMR (100 MHz, d_4_-MeOD) δ 161.8 (d, *J* = 249 Hz, C), 138.7 (C), 131.5 (C), 129.5 (q, *J* = 32 Hz, C), 128.9 (C), 128.0 (2CH), 127.0 (2CH), 125.5 (q, *J* = 4 Hz, 2CH), 124.0 (q, *J* = 272 Hz, CF_3_), 122.4 (CH), 116.8 (d, *J* = 23 Hz, 2CH), 56.3 (CH_2_); 1 C not observed. ^19^F-NMR (376 MHz, d_4_-MeOD) δ −64.2 (3F), δ −113.6 (1F). IR (neat, cm^−1^) 2926 (w), 2842 (m), 2704 (m), 1654 (m), 1614 (m), 1577 (m), 1506 (s), 1319 (s), 1110 (m), 832 (s), 688 (m), 646 (m), 507 (s). HR-MS (TOF-ES+) calculated for C_17_H_12_F_4_N_2_S (M + H) 357.0736, found 353.0742 (Δ = 1.8 ppm).

*(Z)-N-(4-Chloro-2-(trifluoromethyl)phenyl)-5-(4-methylbenzylidene)-4,5-dihydrothiazol-2-amine* (**13d**): Yield: 78% (0.29 g; 0.78 mmol). Appearance: yellow solid. ^1^H-NMR (400 MHz, CDCl_3_) δ 7.59 (s, 1H), 7.41–7.44 (m, 1H), 7.10–7.17 (m, 5H), 6.54 (s, 1H), 4.57 (s, 2H), 2.33 (s, 3H). ^13^C-NMR (100 MHz, CDCl_3_) δ 137.3 (C), 132.6 (C), 132.8 (CH), 129.3 (2CH), 128.5 (C), 127.7 (2CH), 126.6 (q, *J* = 6 Hz, CH), 125.1 (CH), 120.7 (CH), 53.8 (CH_2_), 21.2 (CH_3_), not all C observed. ^19^F-NMR (376 MHz, CDCl_3_) δ −61.9. IR (neat, cm^−1^) 3083 (w), 3045 (w), 2124 (w), 1902 (w), 1764 (w), 1764 (s), 1625 (m), 1597 (m), 1481 (m), 1468 (m), 1371 (w), 1306 (s), 1268 (m), 1250 (m), 1209 (m), 1195 (m), 1119 (s), 1049 (s), 855 (m), 836 (s), 796 (s), 617 (s), 521 (s), 441 (m). HR-MS (TOF-ES+) calculated for C_18_H_14_ClF_3_N_2_S (M + H) 383.0585, found 383.0597 (Δ = −1.2 ppm).

*(Z)-5-(2-Bromobenzylidene)-N-(4-chloro-2-(trifluoromethyl)phenyl)-4,5-dihydrothiazol-2-amine* (**13e**): Yield: 75% (0.34 g; 0.75 mmol). Appearance: yellow solid. ^1^H-NMR (400 MHz, CDCl_3_) δ 7.58 (d, *J* = 2.5 Hz, 1H), 7.55 (dd, *J* = 8.0, 1.2 Hz, 1H), 7.40 (dd, *J* = 8.5, 2.5 Hz, 1H), 7.32 (dd, *J* = 7.8, 1.8 Hz, 1H), 7.30–7.22 (m, 1H), 7.11–7.04 (m, 2H), 6.73 (t, *J* = 2.2 Hz, 1H), 4.59 (d, *J* = 2.2 Hz, 2H). ^13^C-NMR (100 MHz, CDCl_3_) δ 161.0 (C), 146.6 (C), 135.7 (C), 134.3 (C), 132.9 (CH), 132.7 (CH), 128.9 (CH), 128.7 (C), 128.3 (CH), 127.5 (CH), 126.6 (C, q, *J* = 5 Hz), 125.5 (CH), 124.2 (C, q, *J* = 30 Hz), 123.8 (C), 123.2 (CF_3_, q, *J* = 271 Hz), 119.9 (CH), 53.5 (CH_2_). ^19^F-NMR (376 MHz, CDCl_3_) δ −61.8. IR (neat, cm^−1^) 3049 (m), 2898 (m), 1653 (s), 1622 (s), 1483 (s), 1433 (m), 1309 (s), 1193 (m), 1161 (m), 1124 (s), 1101 (m), 1050 (s), 891 (m), 839 (m), 671 (m). HR-MS (TOF-ES+) calculated for C_17_H_12_ClBrF_3_N_2_S (M + H) 446.9545, found 446.9559 (Δ = 3.1 ppm).

*(Z)-N-(4-Chloro-2-(trifluoromethyl)phenyl)-5-(naphthalen-1-ylmethylene)-4,5-dihydro-thiazol-2-amine* (**13f**): Yield: 80% (0.33 g; 0.8 mmol). Appearance: yellow solid. ^1^H-NMR (400 MHz, CDCl_3_) δ 7.97–7.92 (m, 1H), 7.87–7.82 (m, 1H), 7.76 (dq, *J* = 6.9, 3.4 Hz, 1H), 7.55 (d, *J* = 2.4 Hz, 1H), 7.54–7.47 (m, 2H), 7.44–7.40 (m, 2H), 7.36 (dd, *J* = 8.5, 2.4 Hz, 1H), 7.16–7.12 (m, 1H), 7.05 (d, *J* = 8.4 Hz, 1H), 6.61 (s, 1H), 4.69 (s, 2H). ^13^C-NMR (100 MHz, CDCl_3_) δ 133.5 (C), 133.1 (C), 132.6 (CH), 131.1 (C), 128.6 (CH), 128.4 (C), 128.3 (CH), 126.5 (CH, m), 126.3 (CH), 126.1 (CH), 125.4 (CH), 125.3 (CH), 125.1 (CH), 123.8 (CH), 123.8 (C), 118.2 (CH), 53.2 (CH2); several C not observed. ^19^F-NMR (376 MHz, CDCl_3_) δ −61.9. IR (neat, cm^−1^) 2982 (w), 1627 (m), 1590 (m), 1539 (m), 1484 (m), 1419 (m), 1309 (s), 1290 (m), 1174 (m), 1119 (s), 1050 (s), 795 (m), 686 (m), 641 (m). HR-MS (TOF-ES+) calculated for C_21_H_15_ClF_3_N_2_S (M + H) 419.0597, found 419.0610 (Δ = 3.2 ppm).

## 4. Conclusions

In conclusion, we have developed a straightforward access to valuable propargylic amines that exploits a telescoped continuous flow approach. This sequence features the safe use of DPPA as the azide source for converting propargylic alcohols into the intermediate azide counterparts. A Staudinger reduction process was used as part of the same sequence to yield the desired propargylic amine species in good yield and purity. Final optimization of this telescoped flow sequence furthermore led to using the more sustainable 2-MeTHF as solvent as opposed to THF and included an in-line extractive work-up. The value of generating these amine products on demand was demonstrated in their effective transformation into drug-like thiazolines, whose correct structural assignment was accomplished by means of a set of single crystal X-ray structures. We believe that this effective entry into propargylic amines will facilitate their further exploitation in future synthesis programs yielding valuable chemical entities.

## Supplementary Materials

The following are available online: ^1^H and ^13^C-NMR spectra of products where an isolated yield is reported.
