# Supplementary material for: Development of a Telescoped Flow Process for the Safe and Effective Generation of Propargylic Amines
Source: Molecules. 2019 Oct 10;24(20):3658. doi: 10.3390/molecules24203658 (PMC6833020; doi:10.3390/molecules24203658)

Supporting Information

Development of a Telescoped Flow Process for the Safe and Effective Generation of Propargylic Amines

Kian Donnelly,[a] Huan Zhang,[a] Marcus Baumann*[a]

[a] School of Chemistry, University College Dublin, Science Centre South, Belfield, Dublin 4, Ireland

email: marcus.baumann@ucd.ie

Table of contents:

Copies of NMR spectra SI 2

Copies of NMR Spectra

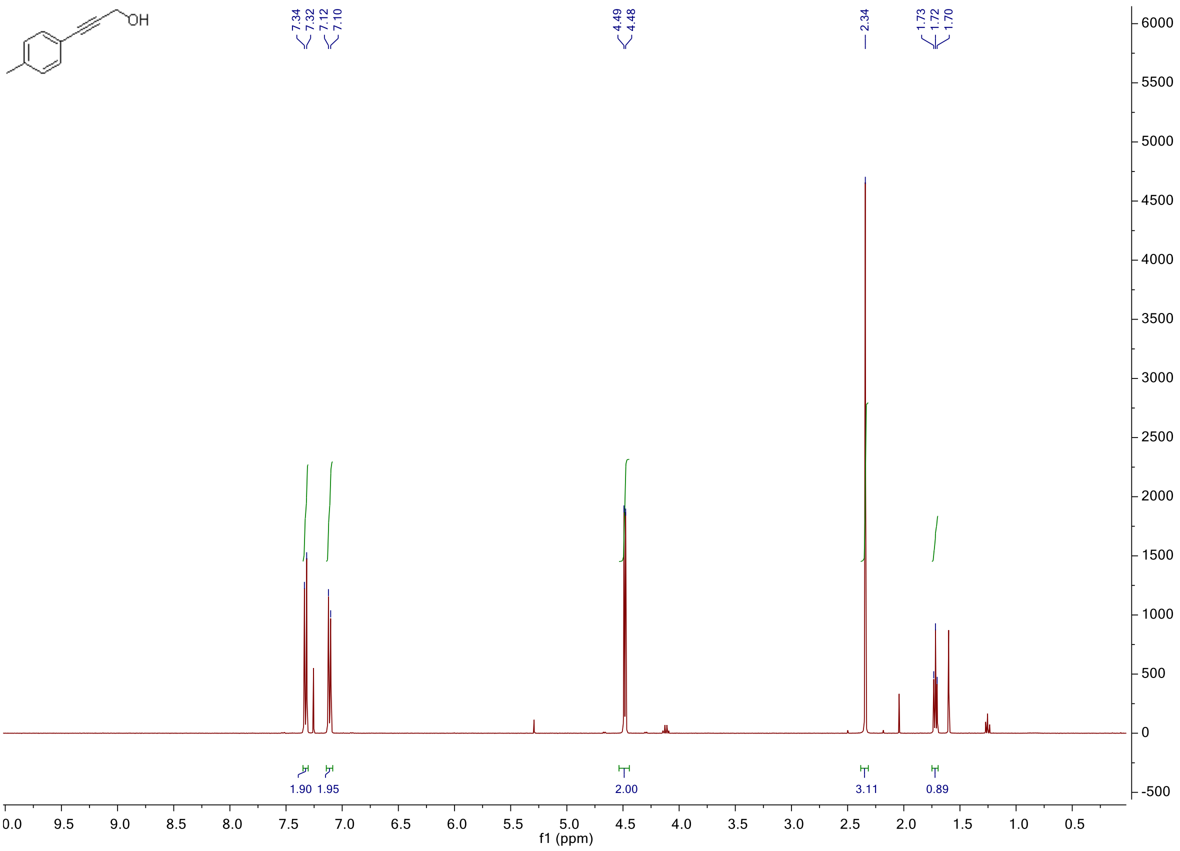

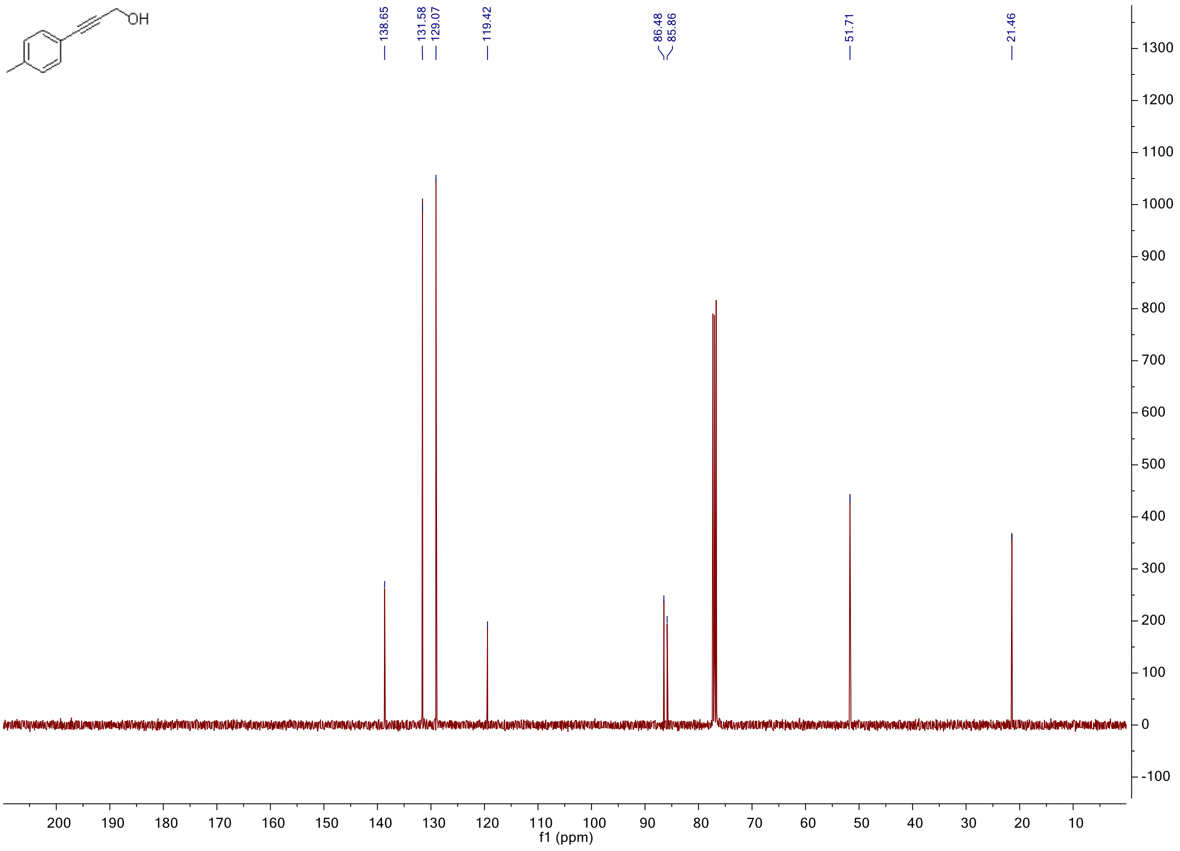

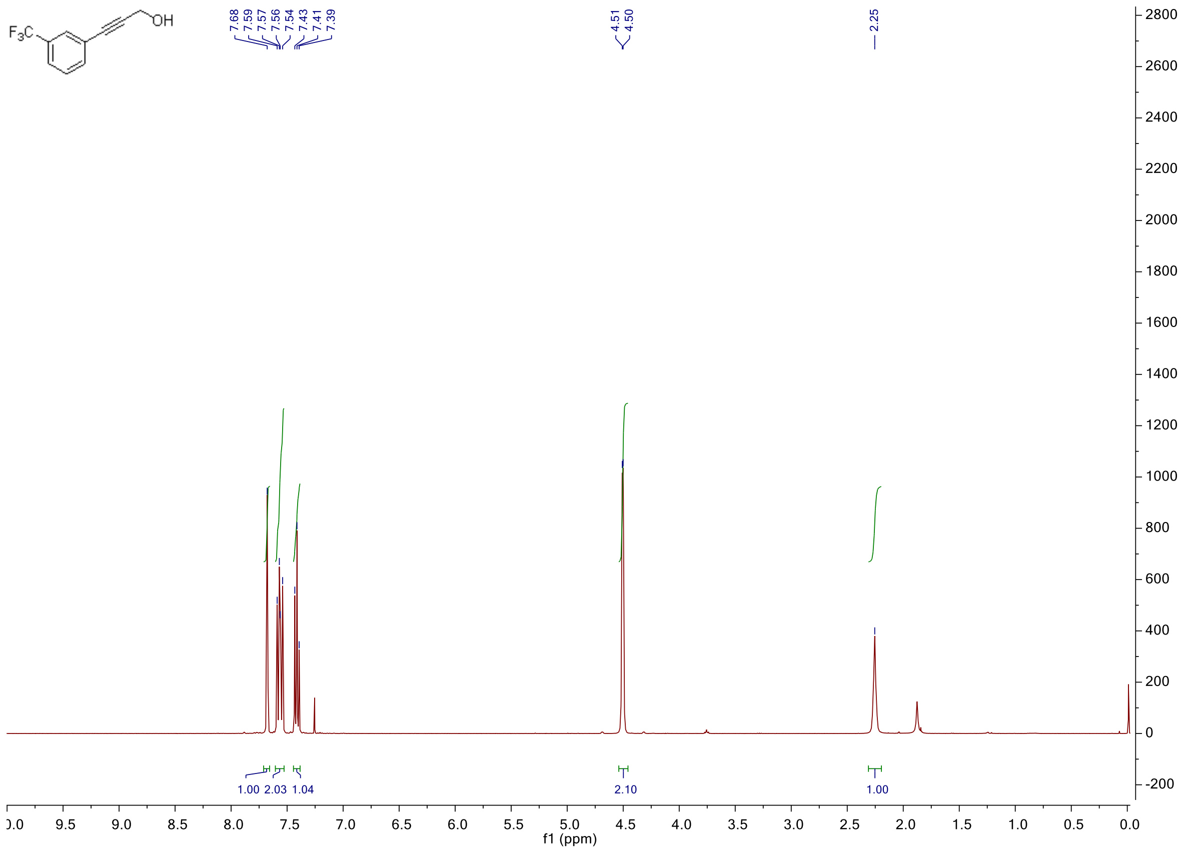
F3C
OH
7.68
7.59
7.57
7.56
7.54
7.43
7.41
7.39
4.51
4.50
2.25
1.00 2.03 1.04
2.10
1.00
f1 (ppm)

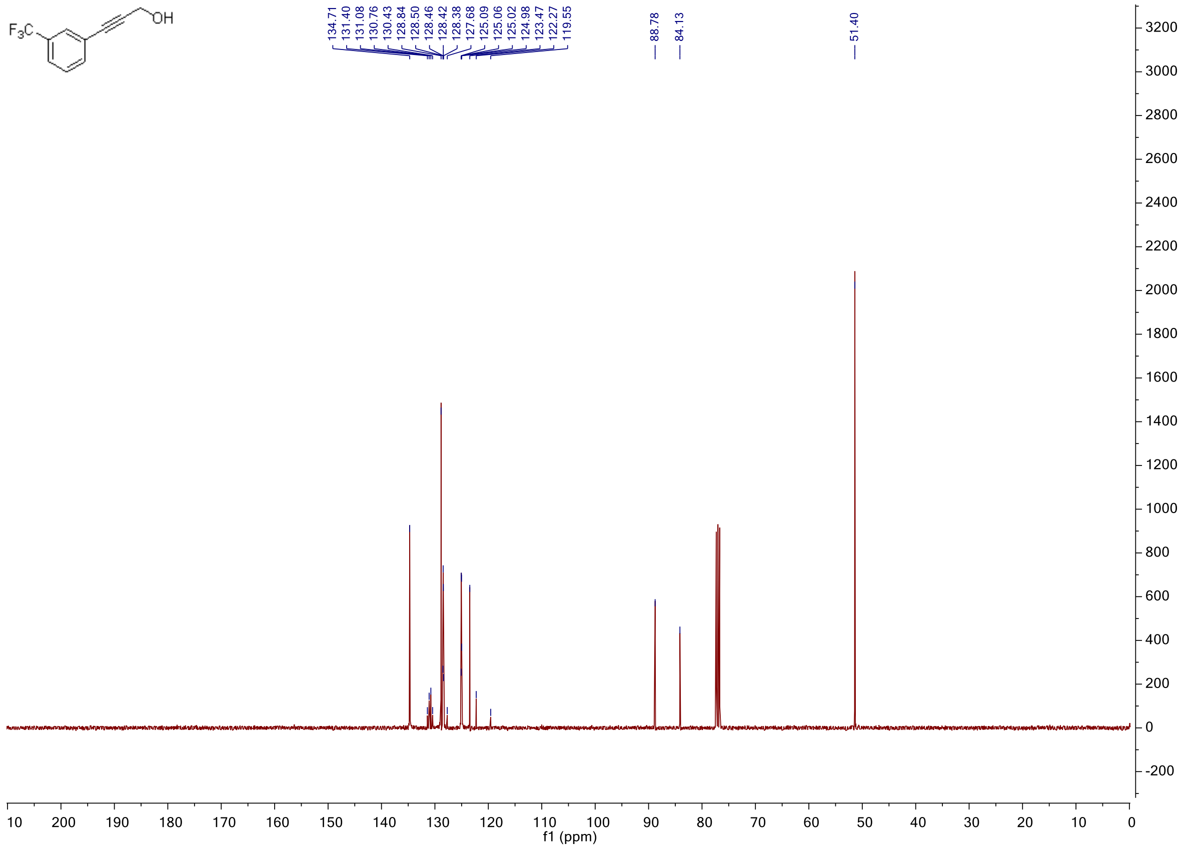
F3C
OH
134.71
131.40
131.08
130.76
130.43
128.84
128.50
128.46
128.42
128.38
127.68
125.09
125.06
125.02
124.98
123.47
122.27
119.55
88.78
84.13
51.40
f1 (ppm)

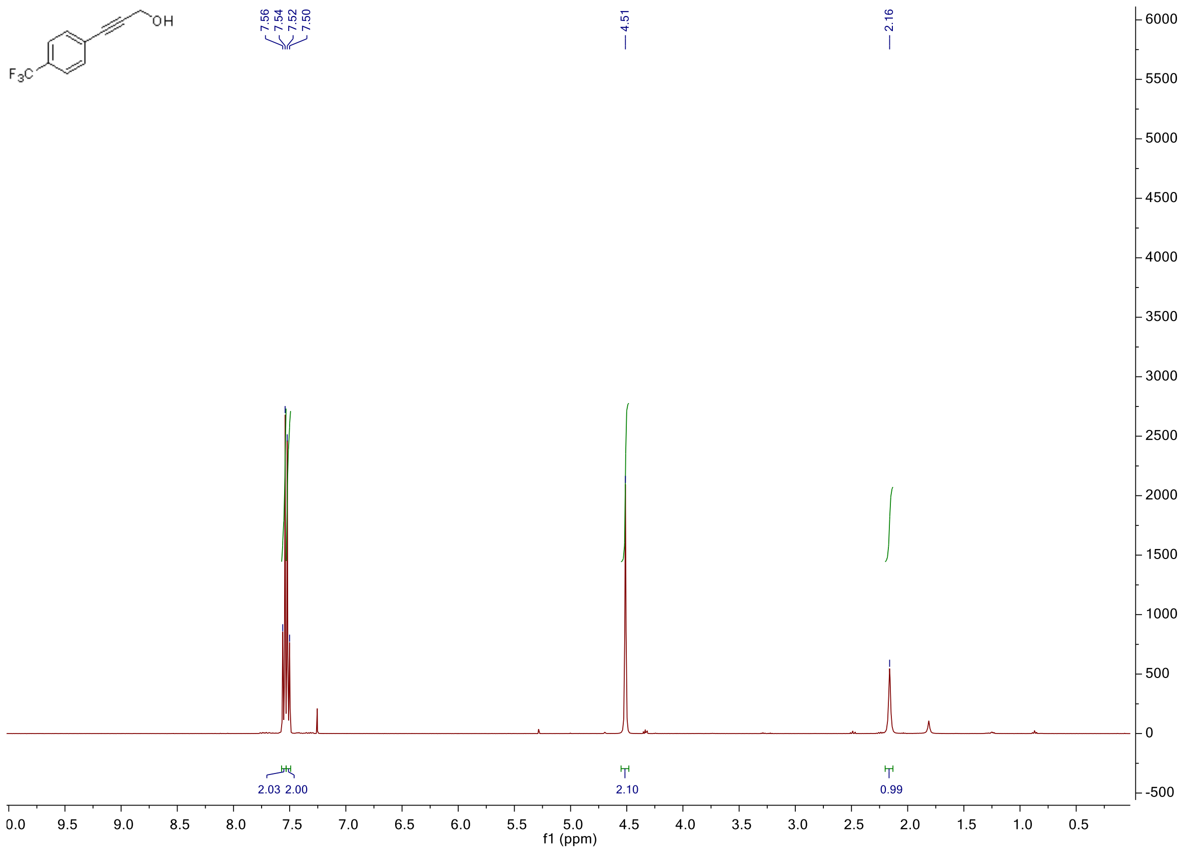
OH
F3C
7.56
7.54
7.52
7.50
4.51
2.16
2.03
2.00
2.10
0.99
f1 (ppm)

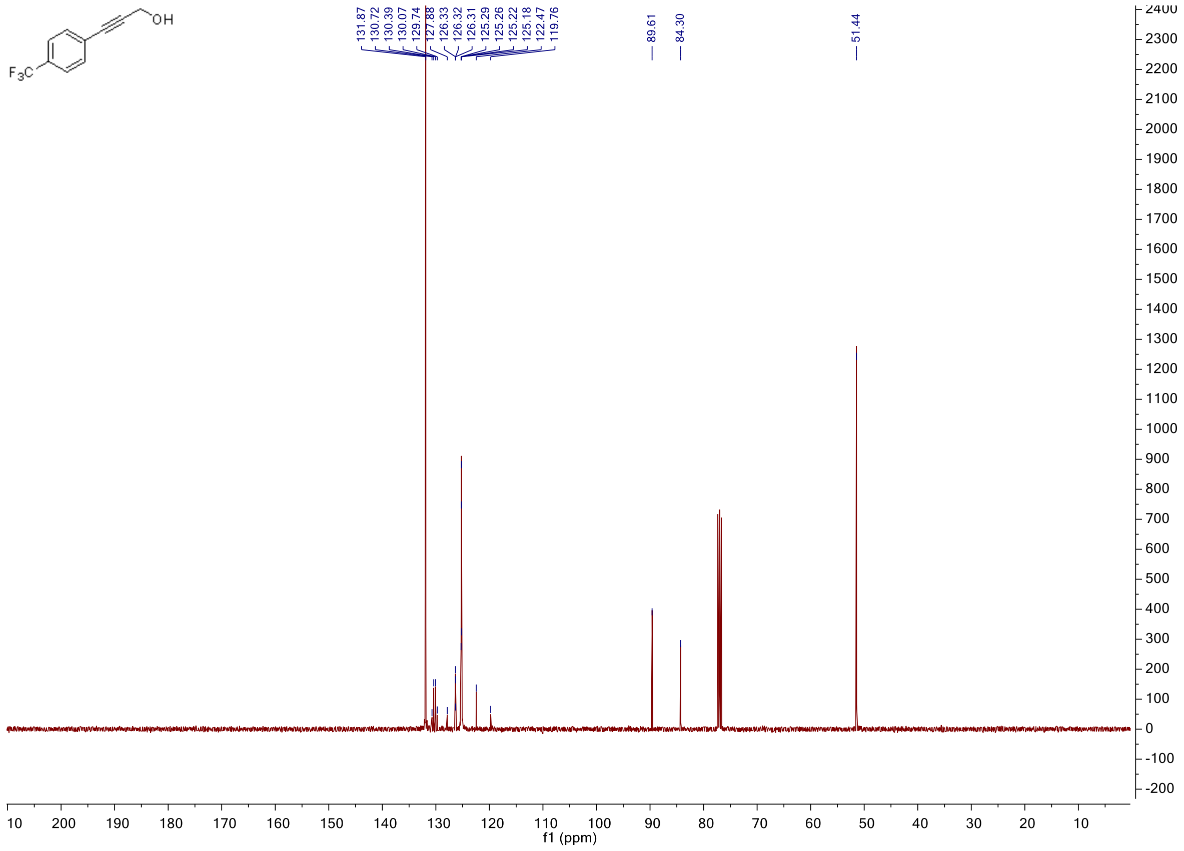
OH
F3C
131.87
130.72
130.39
130.07
129.74
127.88
126.33
126.32
126.31
125.29
125.26
125.22
125.18
122.47
119.76
89.61
84.30
51.44
f1 (ppm)

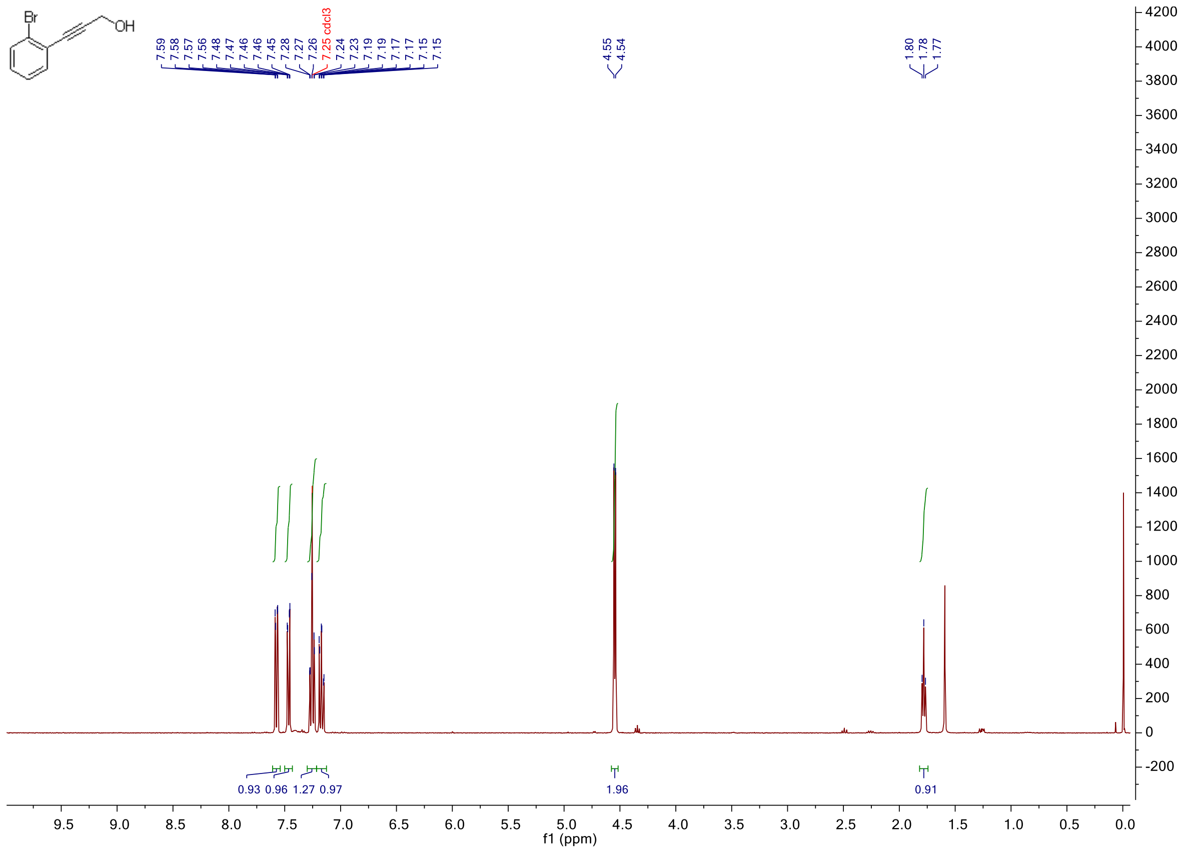
Br
OH
7.59
7.58
7.57
7.56
7.48
7.47
7.46
7.46
7.45
7.28
7.27
7.26
7.25 cdcl3
7.24
7.23
7.19
7.19
7.17
7.17
7.15
7.15
4.55
4.54
1.80
1.78
1.77
0.93 0.96 1.27 0.97
1.96
0.91
f1 (ppm)

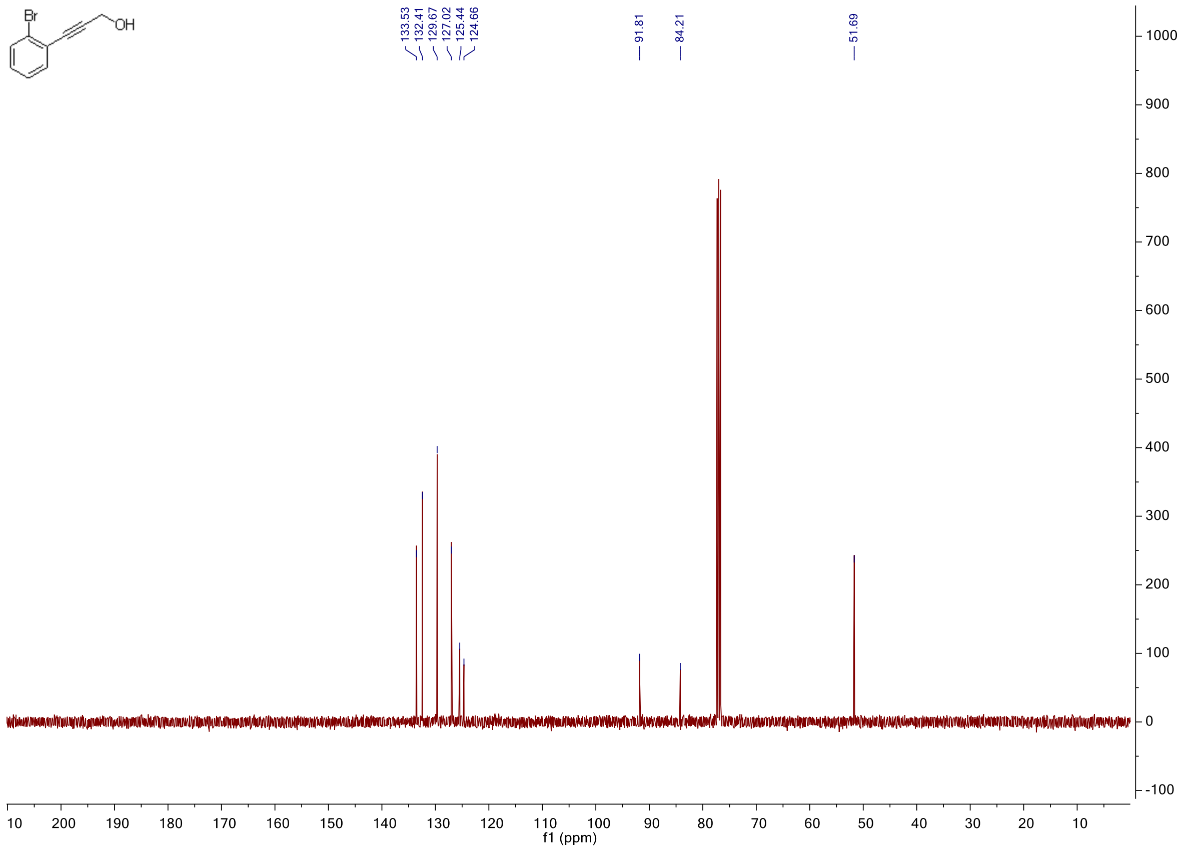
Br
OH
133.53
132.41
129.67
127.02
125.44
124.66
91.81
84.21
51.69
f1 (ppm)

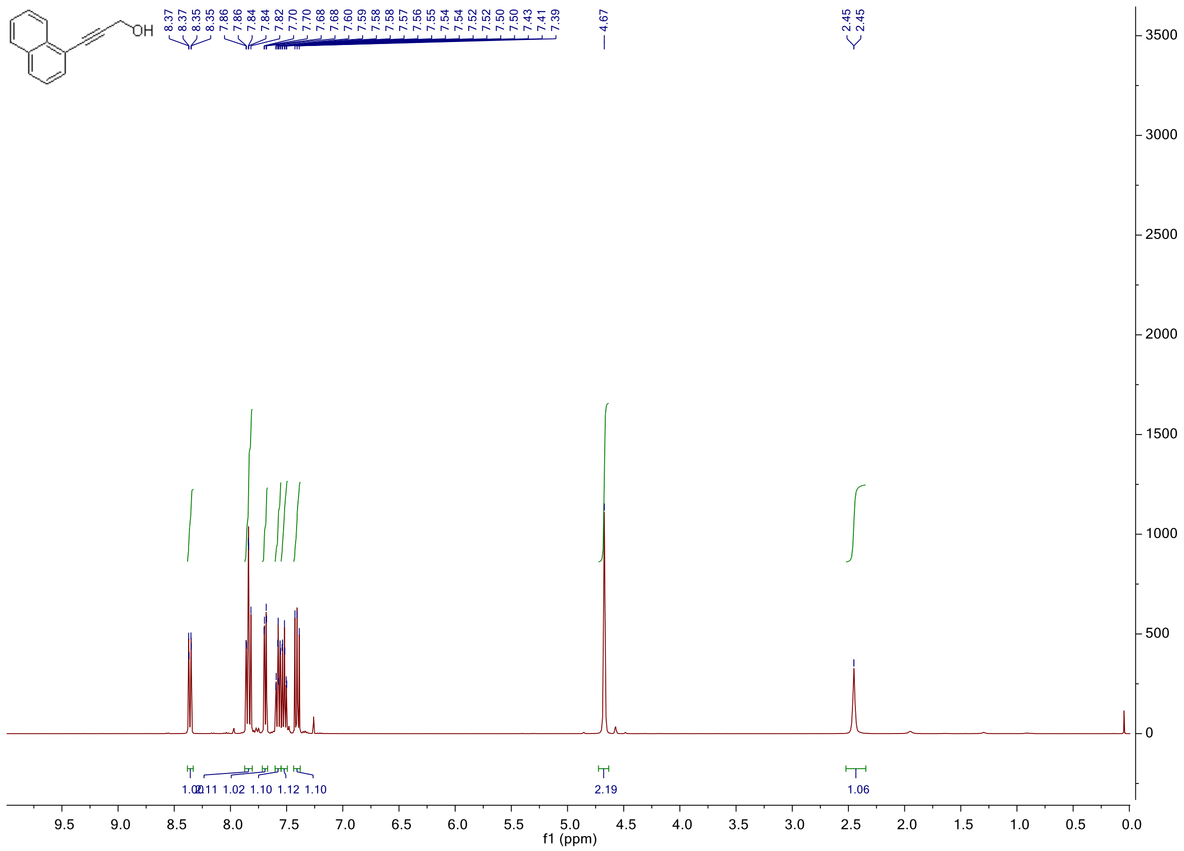

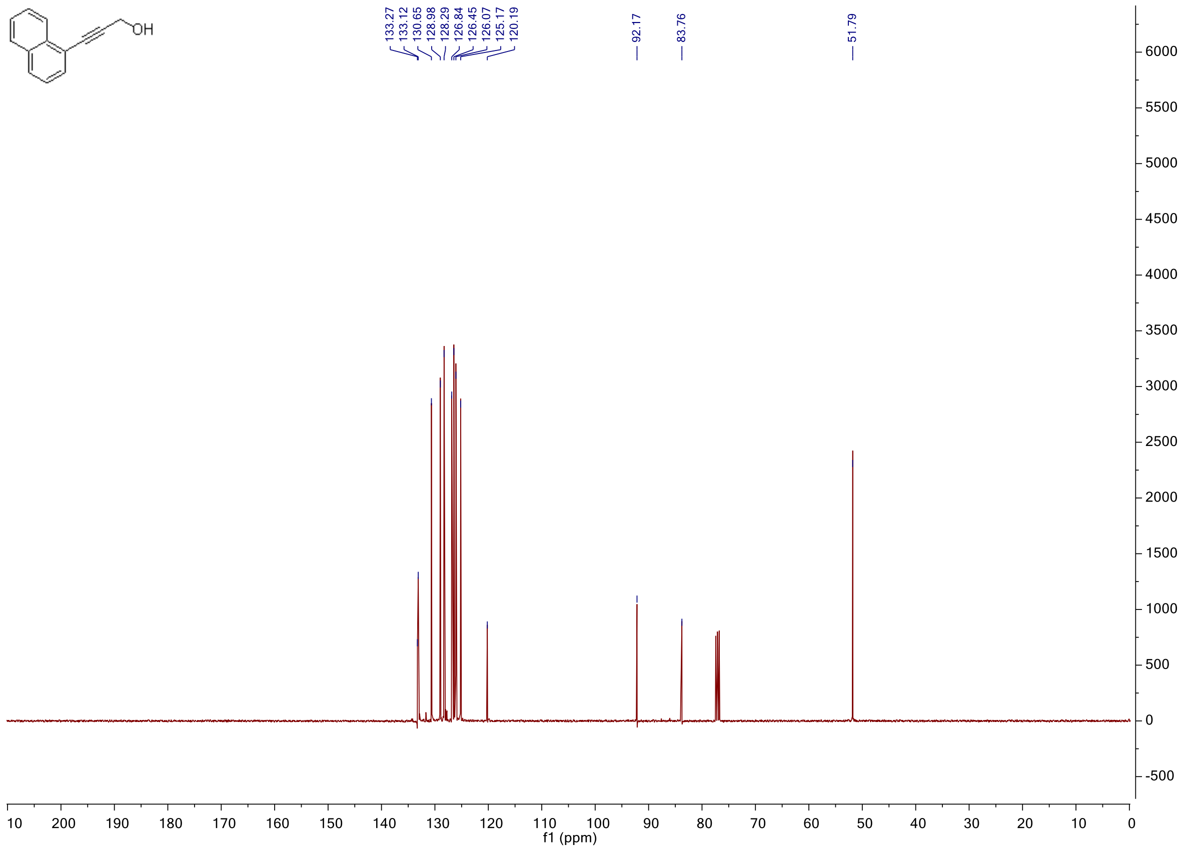

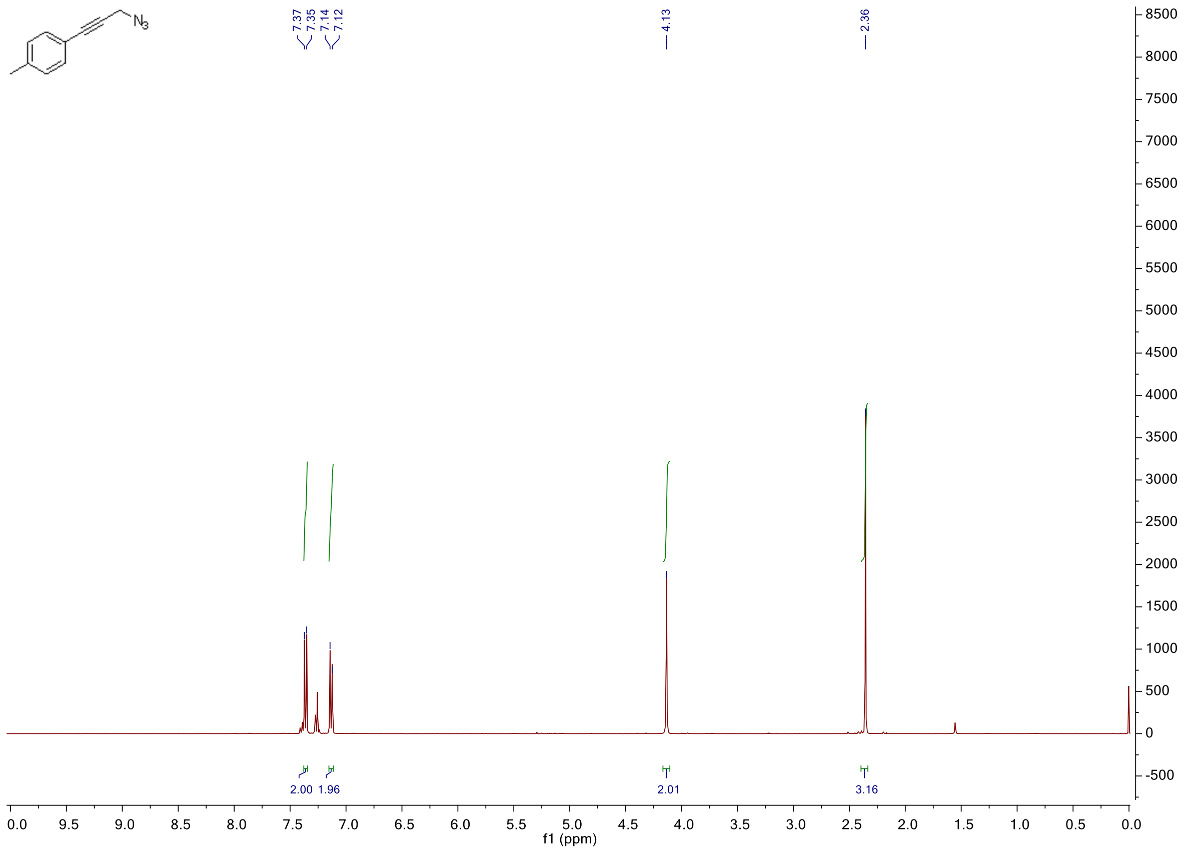
N3
7.37
7.35
7.14
7.12
4.13
2.36
2.00
1.96
2.01
3.16
f1 (ppm)

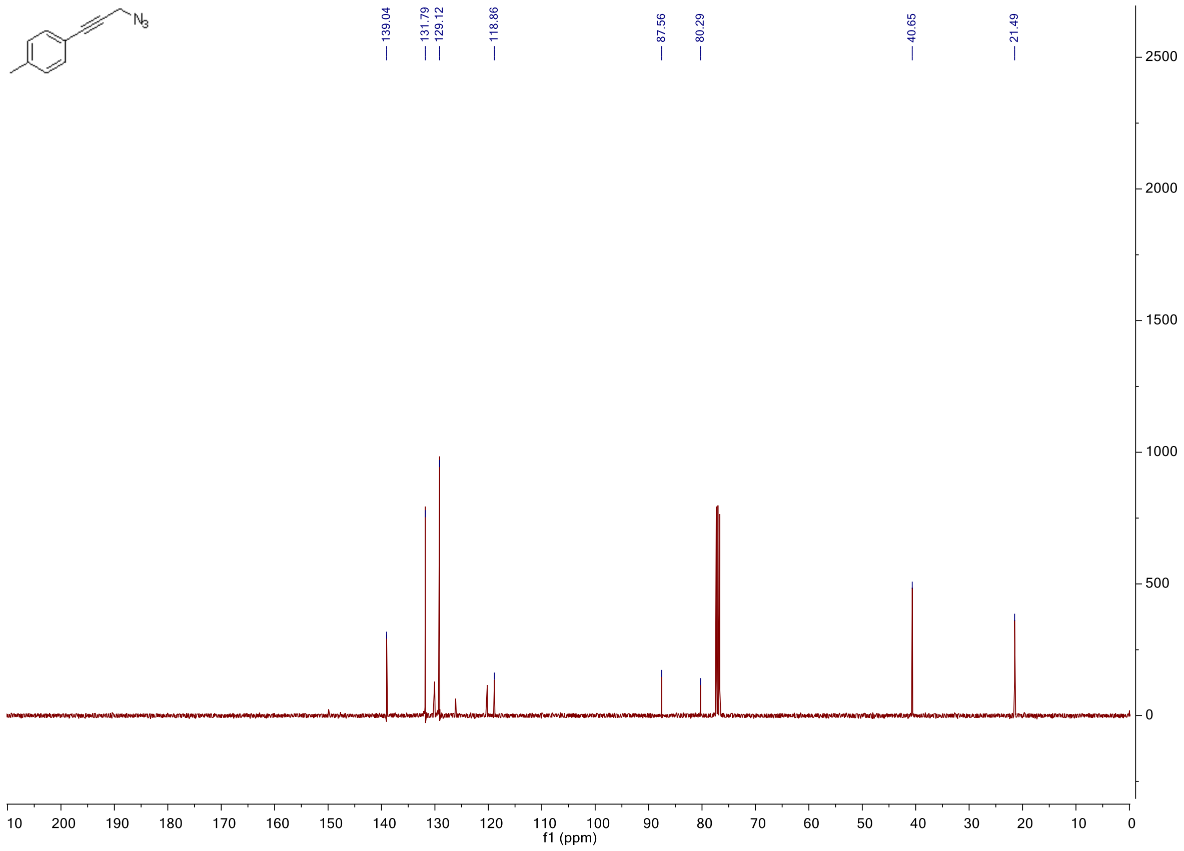
N3
139.04
131.79
129.12
118.86
87.56
80.29
40.65
21.49
f1 (ppm)

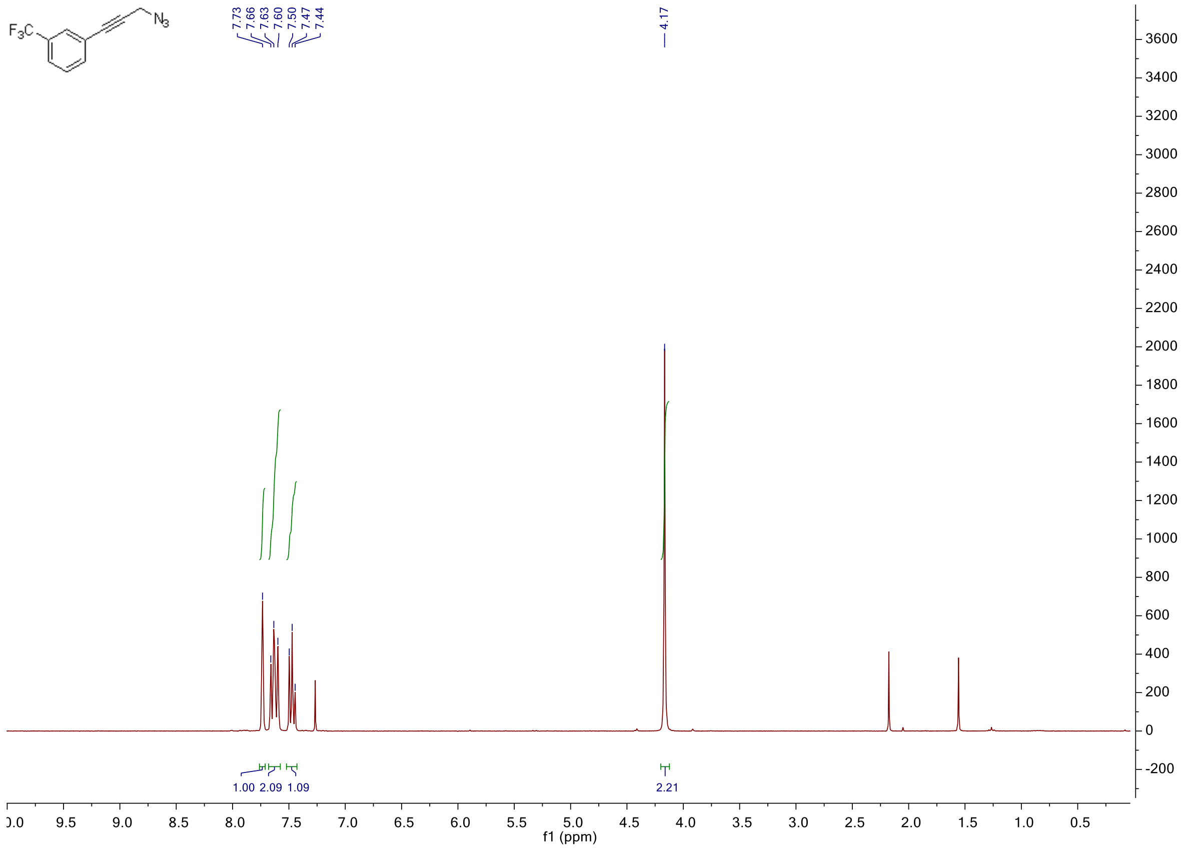

F_3C
N_3
7.73
7.66
7.63
7.60
7.50
7.47
7.44
4.17
1.00
2.09
1.09
2.21
f1 (ppm)

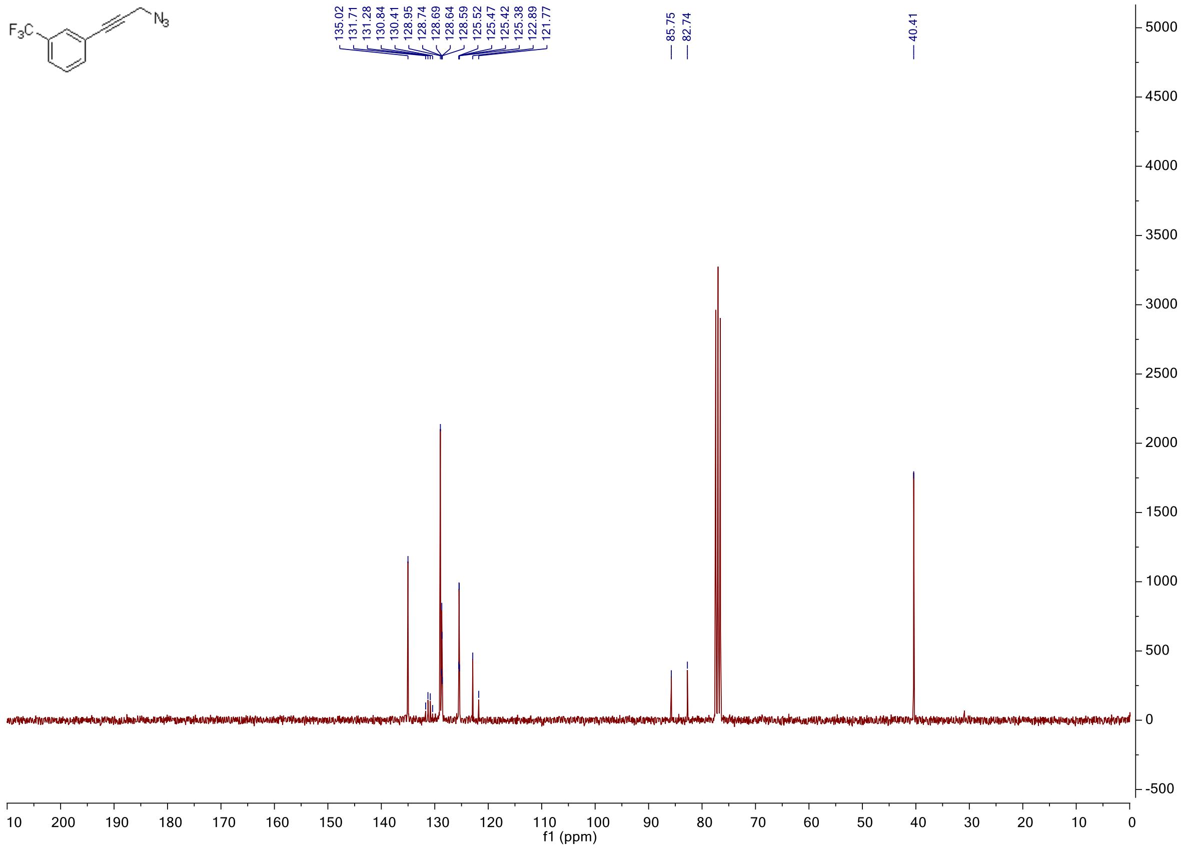

F_3C
N_3
135.02
131.71
131.28
130.84
130.41
128.95
128.74
128.69
128.64
128.59
125.52
125.47
125.42
125.38
122.89
121.77
85.75
82.74
40.41
f1 (ppm)

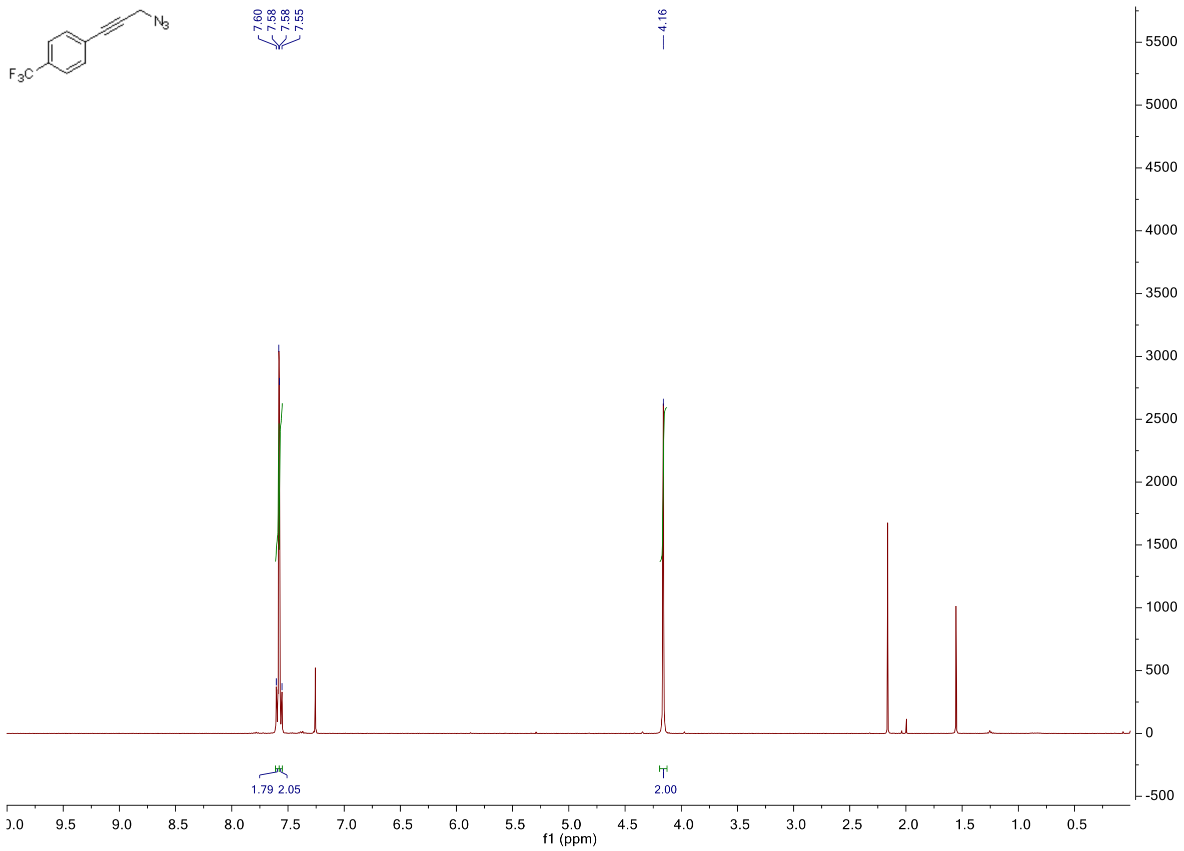

F3C
N3
7.60
7.58
7.58
7.55
4.16
1.79 2.05
2.00
f1 (ppm)

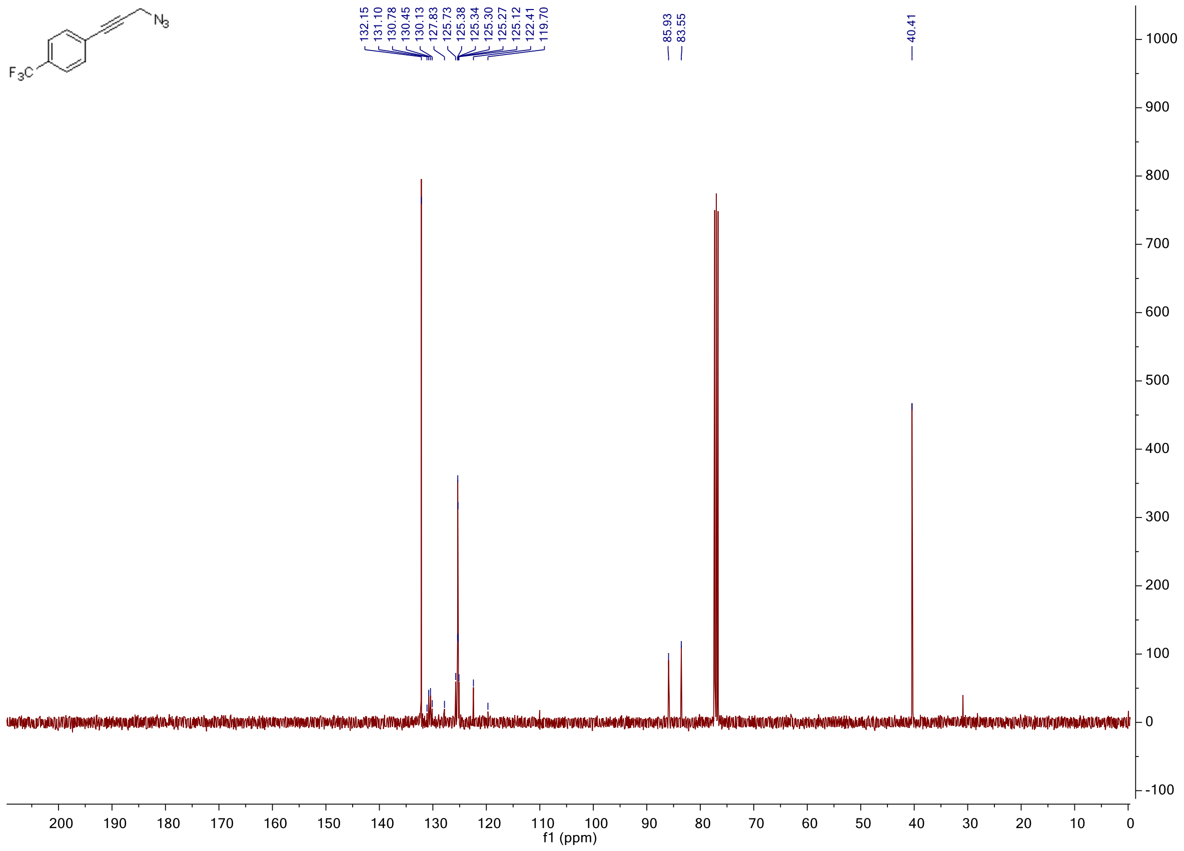

F3C
N3
132.15
131.10
130.78
130.45
130.13
127.83
125.73
125.38
125.34
125.30
125.27
125.12
122.41
119.70
85.93
83.55
40.41
f1 (ppm)

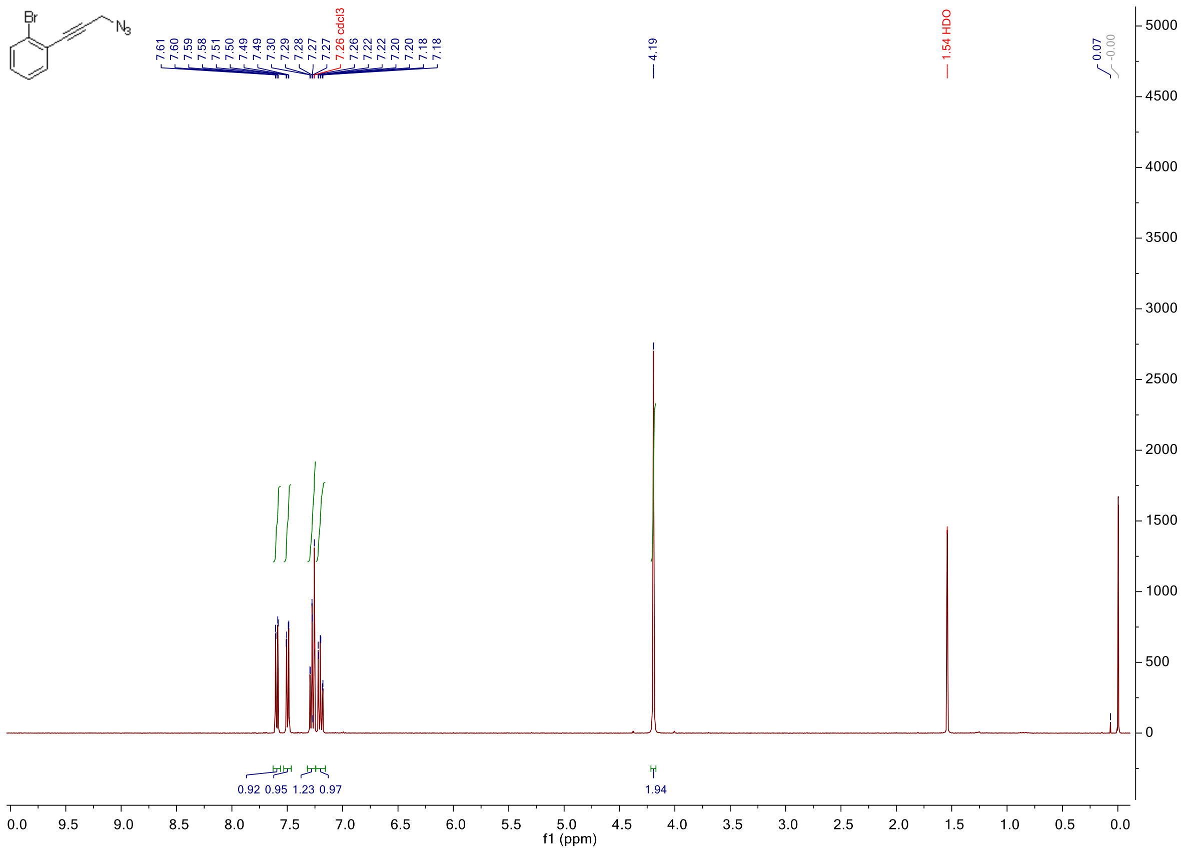
Br
N3
7.61 7.60 7.59 7.58 7.51 7.50 7.49 7.49 7.30 7.29 7.28 7.27 7.27 7.26 cdcl3 7.26 7.22 7.22 7.20 7.20 7.18 7.18
4.19
1.54 HDO
0.07
-0.00
0.92 0.95 1.23 0.97
1.94
f1 (ppm)

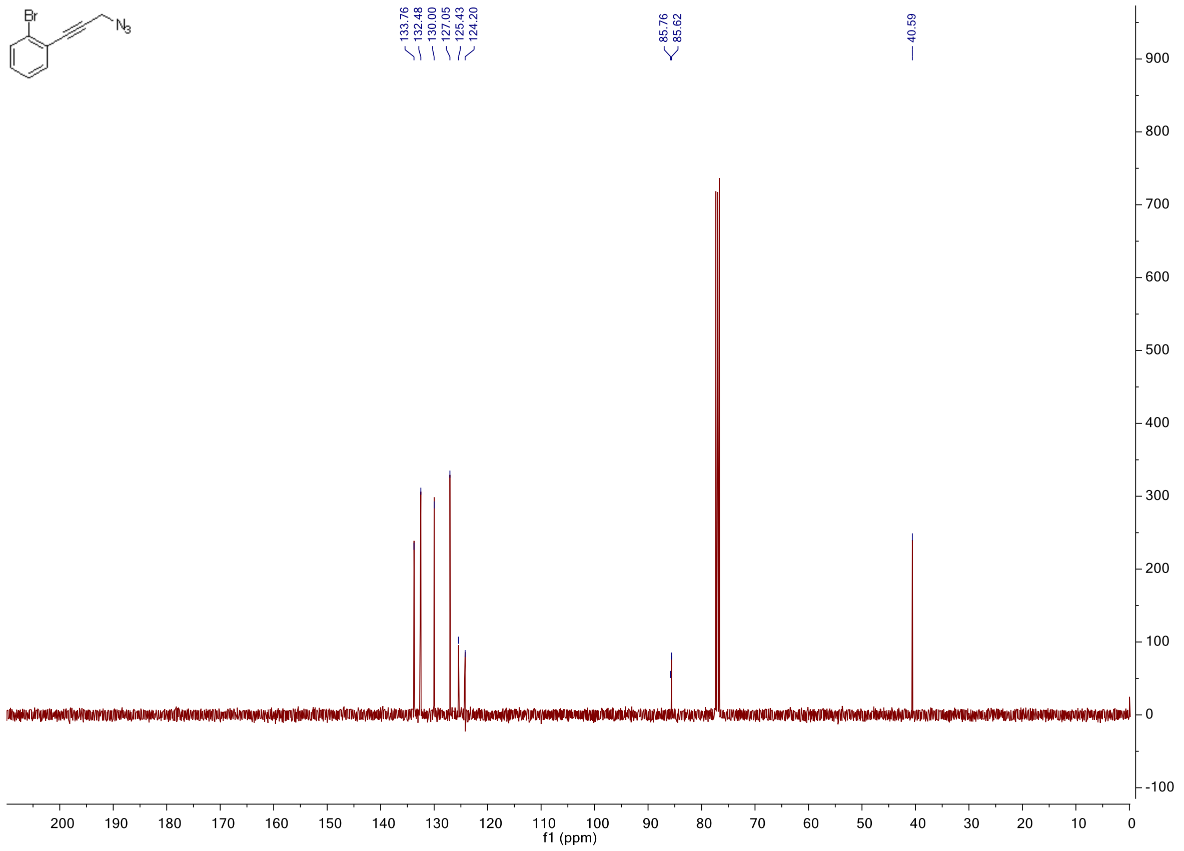
Br
N3
133.76 132.48 130.00 127.05 125.43 124.20
85.76 85.62
40.59
f1 (ppm)

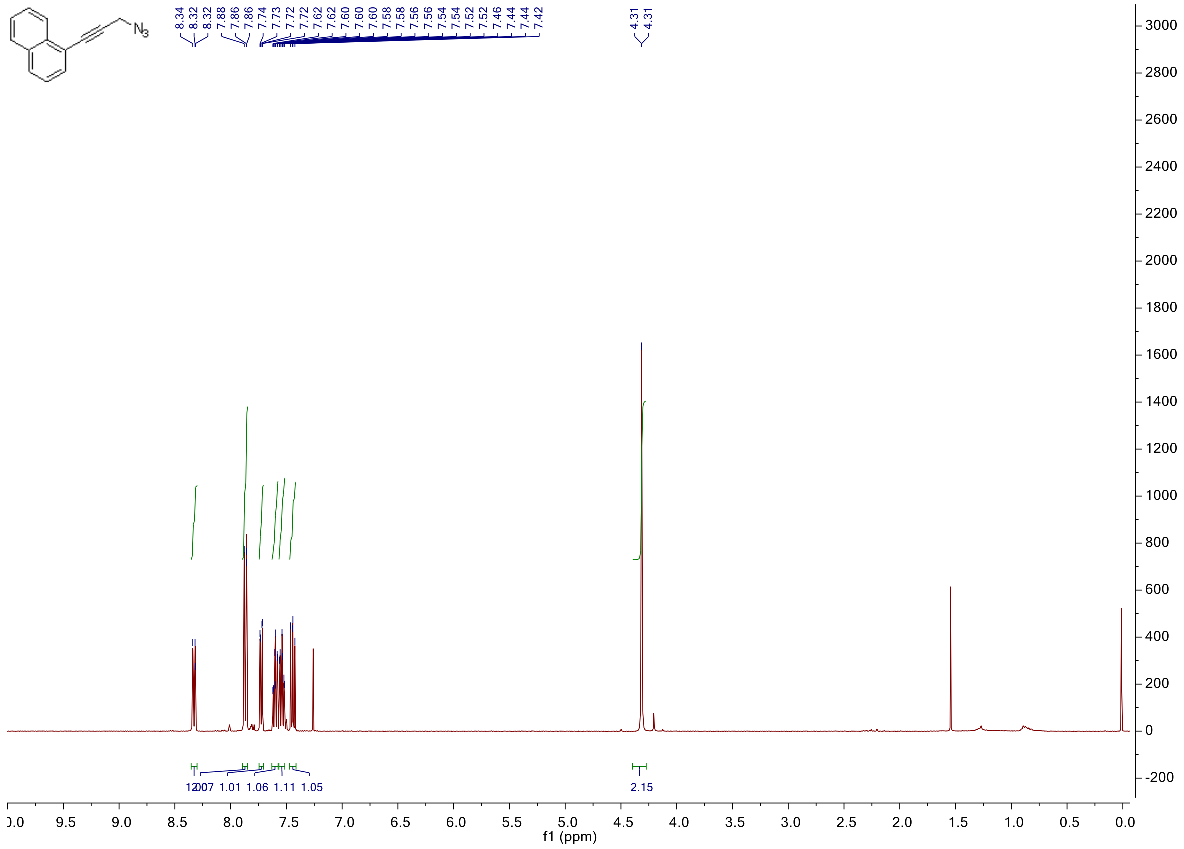
N3
8.34
8.32
8.32
7.88
7.86
7.86
7.74
7.73
7.72
7.72
7.62
7.62
7.60
7.60
7.58
7.58
7.56
7.56
7.54
7.54
7.52
7.52
7.46
7.44
7.44
7.42
4.31
4.31
1.07
1.01
1.06
1.11
1.05
2.15
f1 (ppm)

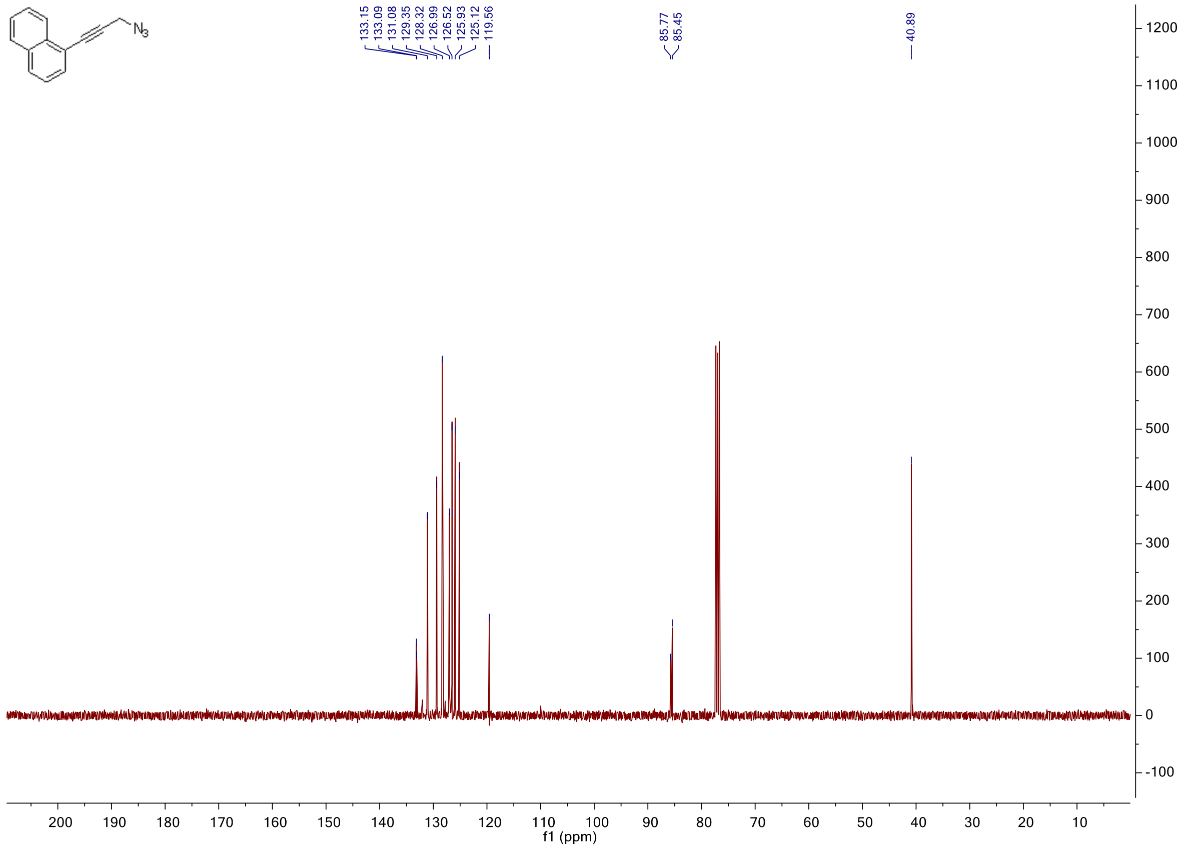
N3
133.15
133.09
131.08
129.35
128.32
126.99
126.52
125.93
125.12
119.56
85.77
85.45
40.89
f1 (ppm)

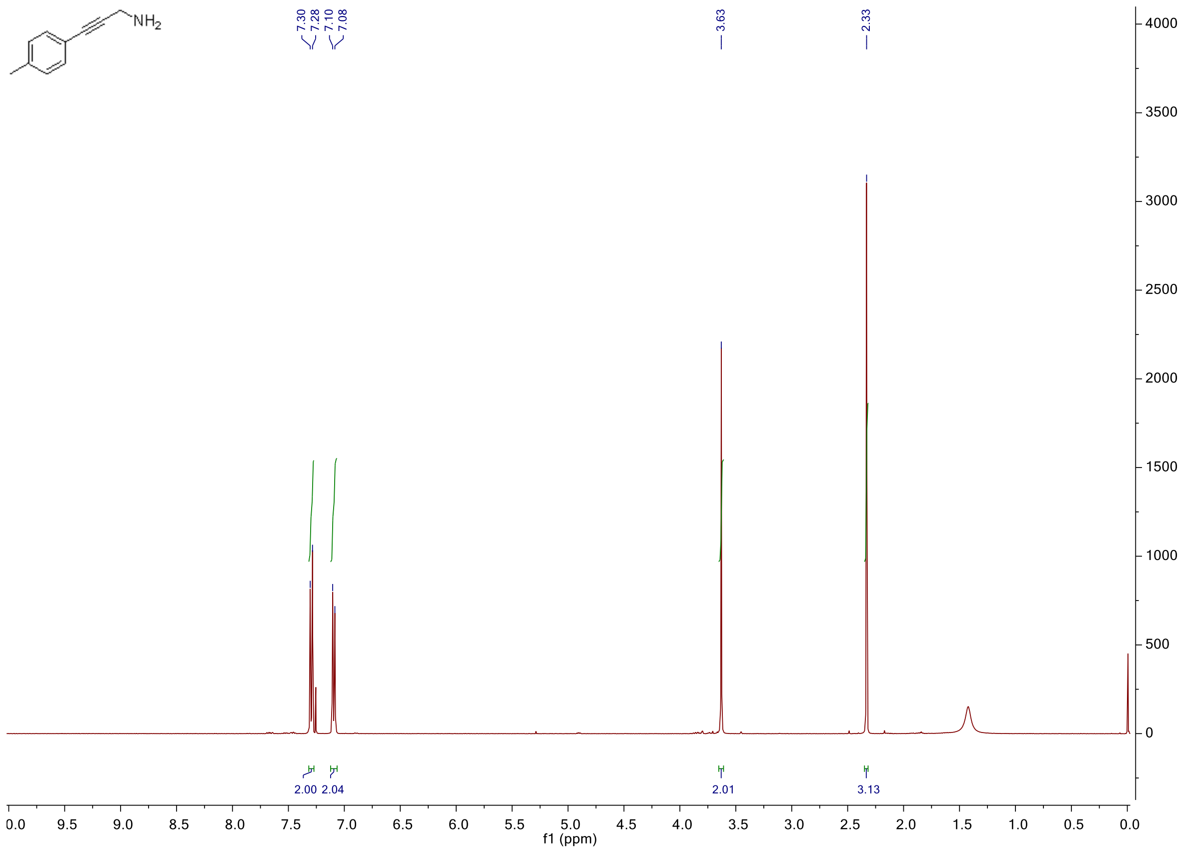
NH2
7.30
7.28
7.10
7.08
3.63
2.33
2.00
2.04
2.01
3.13
f1 (ppm)

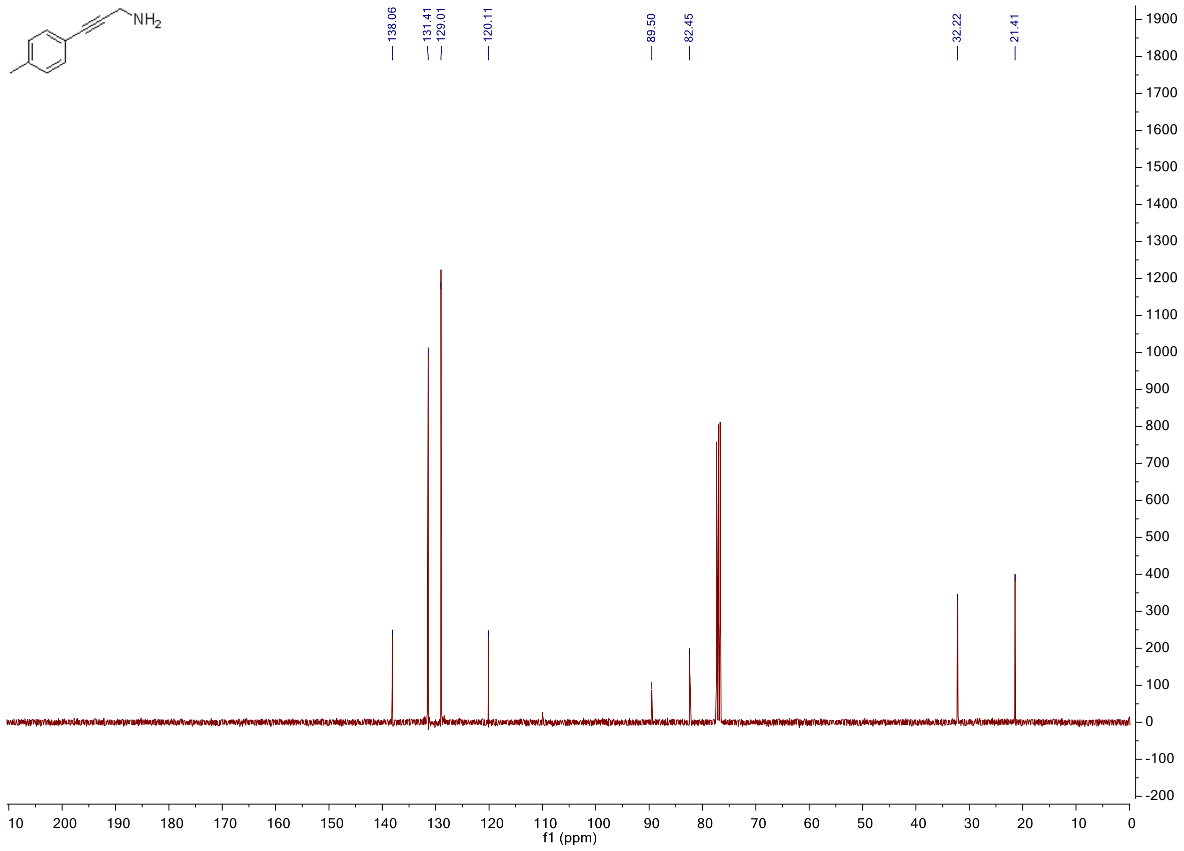
NH2
138.06
131.41
129.01
120.11
89.50
82.45
32.22
21.41
f1 (ppm)

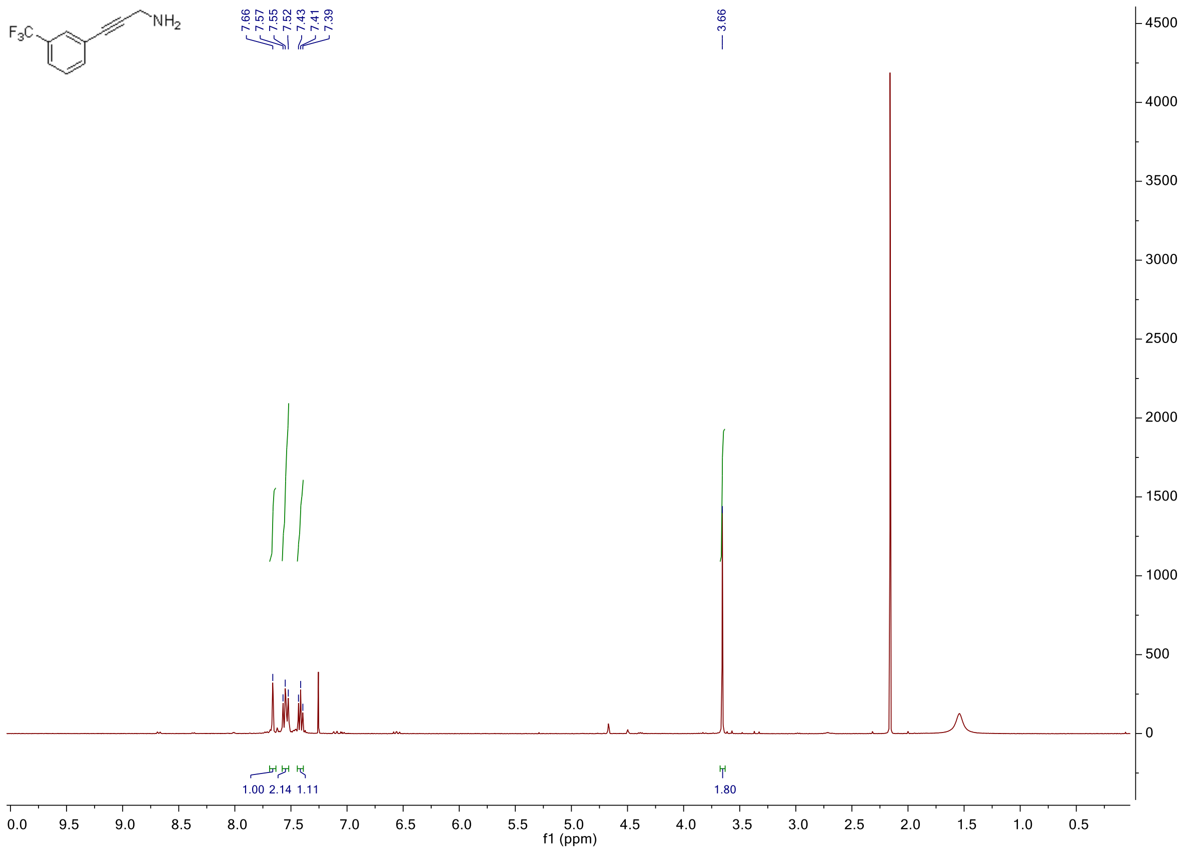

F_3C
NH_2
7.66
7.57
7.55
7.52
7.43
7.41
7.39
3.66
1.00 2.14 1.11
1.80
f1 (ppm)

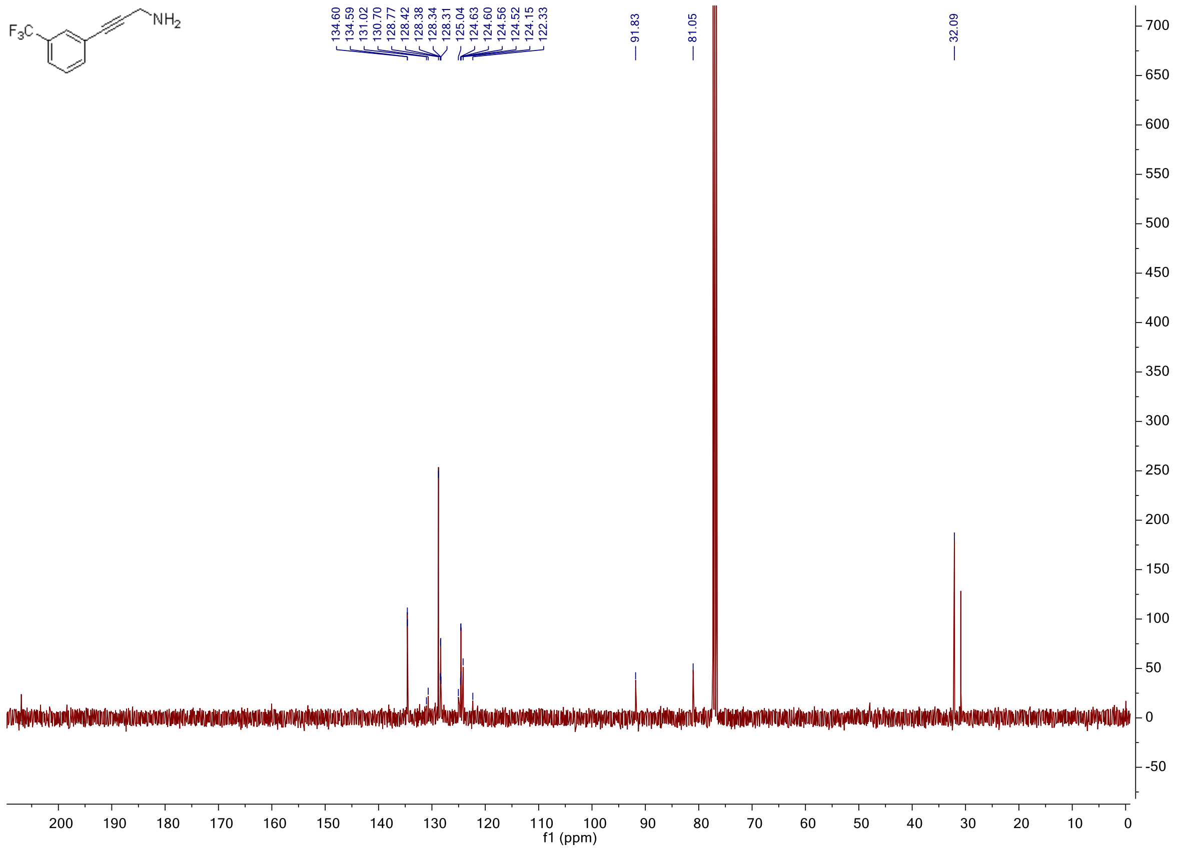

F_3C
NH_2
134.60
134.59
131.02
130.70
128.77
128.42
128.38
128.34
128.31
125.04
124.63
124.60
124.56
124.52
124.15
122.33
91.83
81.05
32.09
f1 (ppm)

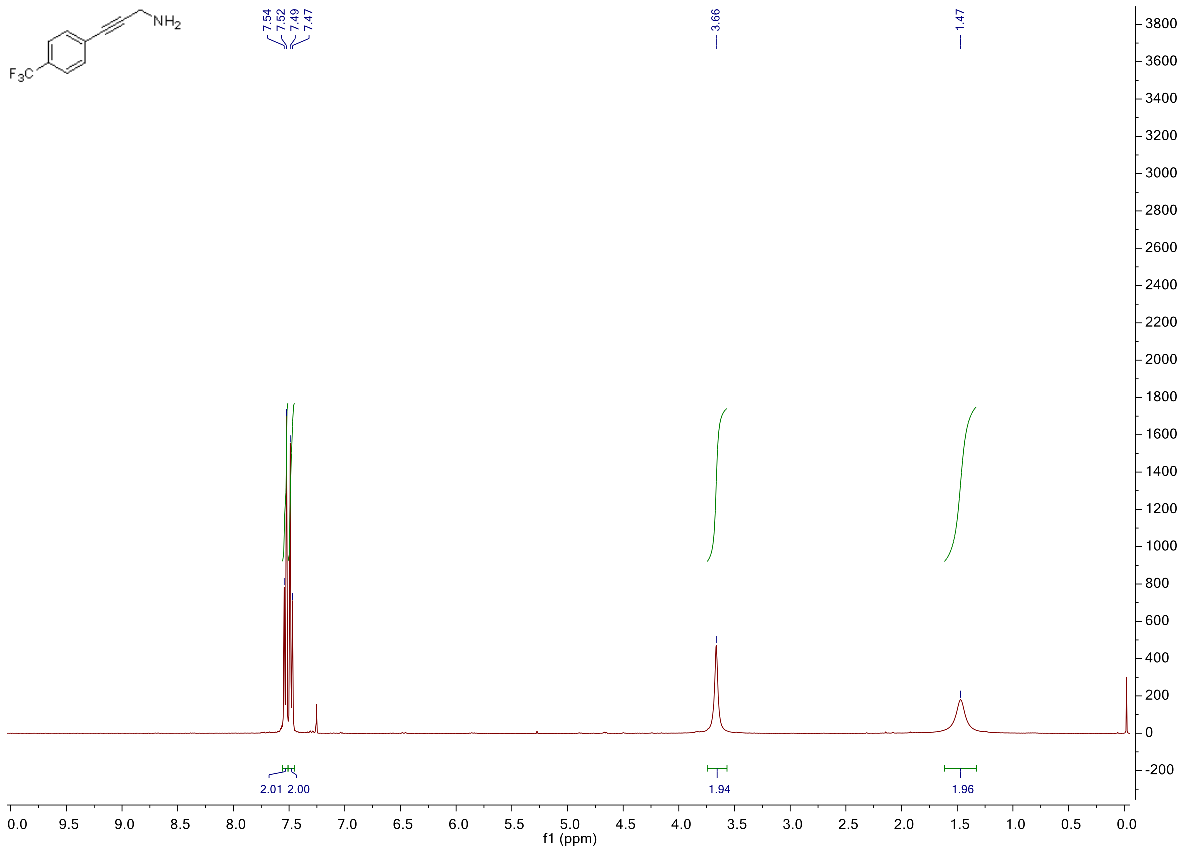
NH2
F3C
7.54
7.52
7.49
7.47
3.66
1.47
2.01
2.00
1.94
1.96
f1 (ppm)

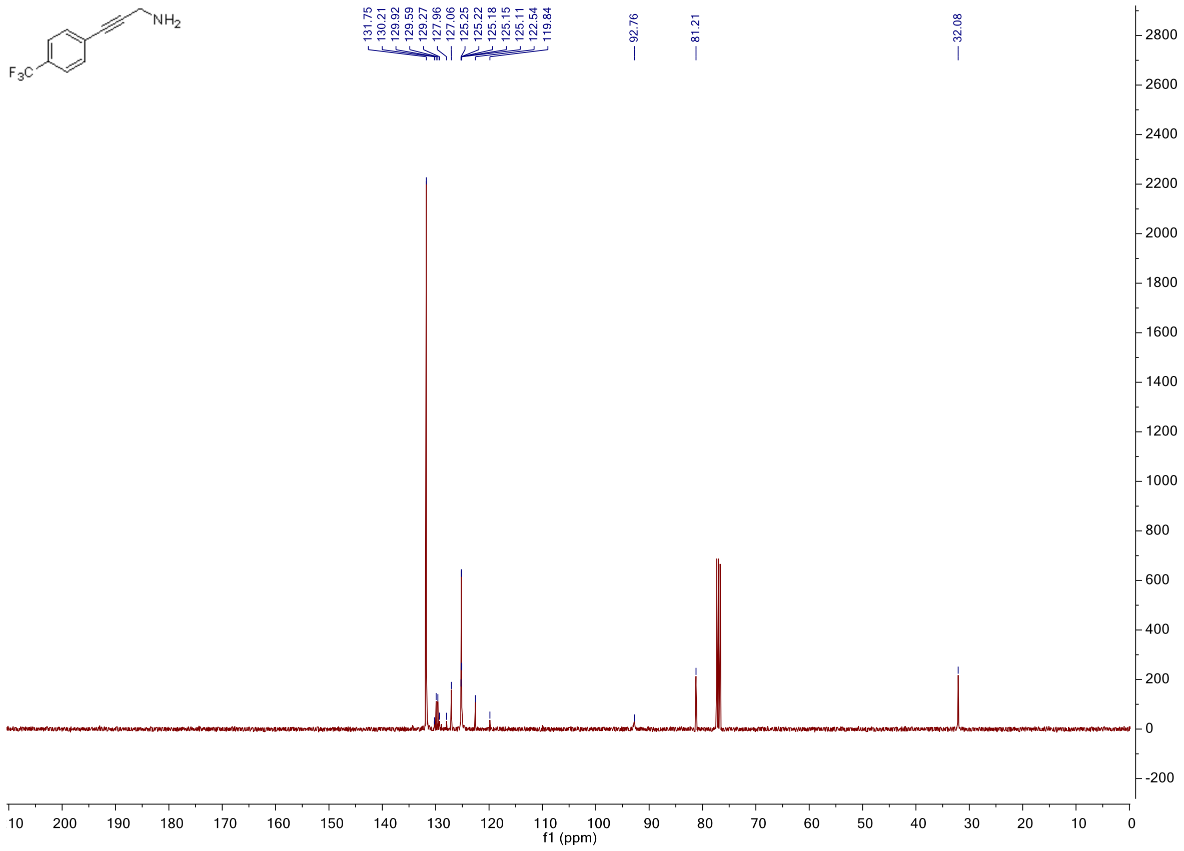
NH2
F3C
131.75
130.21
129.92
129.59
129.27
127.96
127.06
125.25
125.22
125.18
125.15
125.11
122.54
119.84
92.76
81.21
32.08
f1 (ppm)

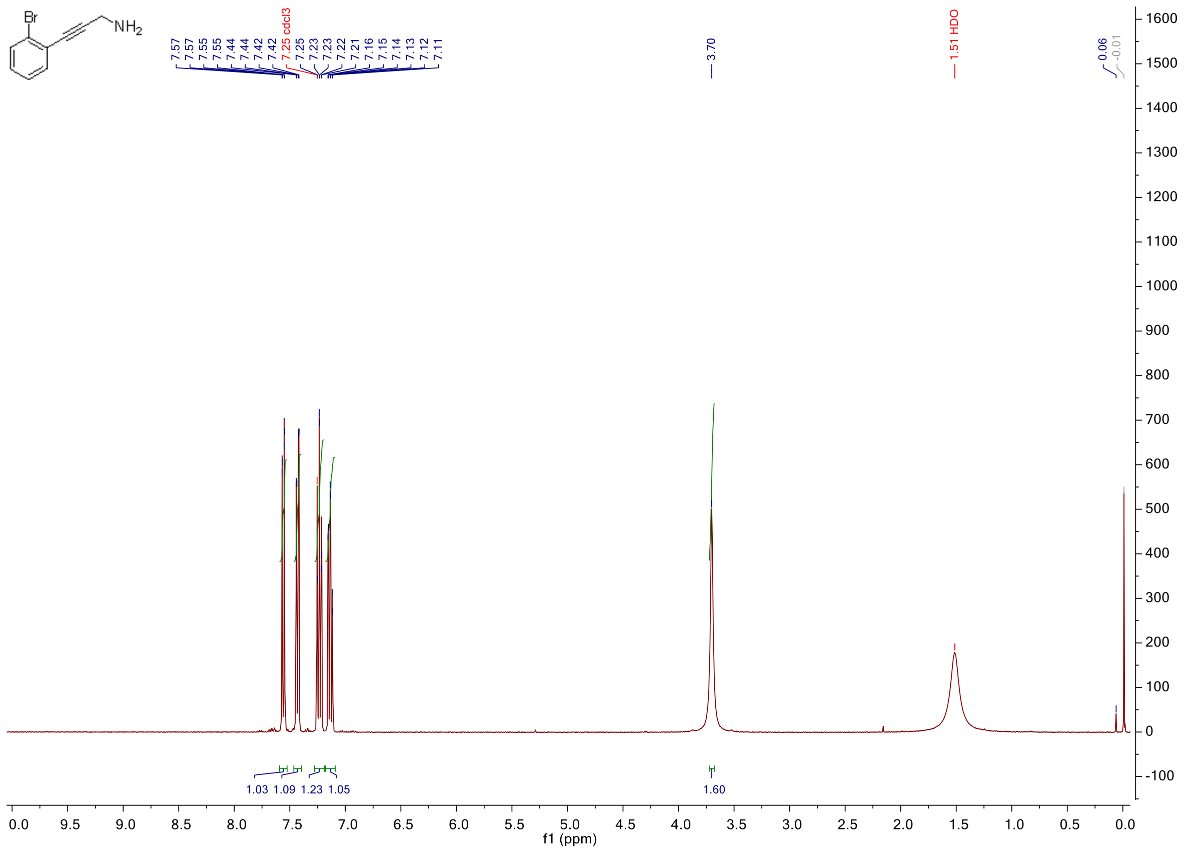

Br
NH2
7.57 7.57 7.55 7.55 7.44 7.44 7.42 7.42 7.25 cdcl3 7.25 7.23 7.23 7.23 7.22 7.21 7.16 7.15 7.14 7.13 7.12 7.11
3.70
1.51 HDO
0.06
-0.01
1.03 1.09 1.23 1.05
1.60
f1 (ppm)

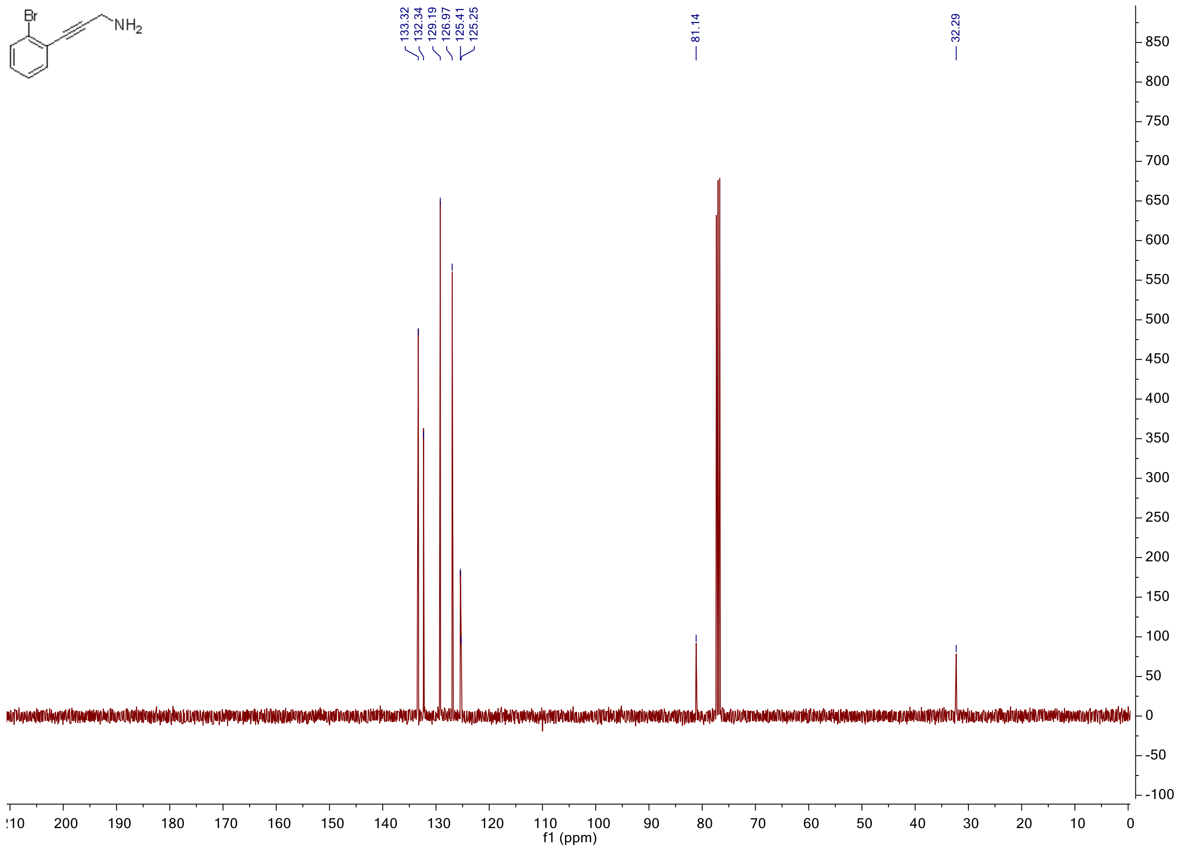

Br
NH2
133.32 132.34 129.19 126.97 125.41 125.25
81.14
32.29
f1 (ppm)

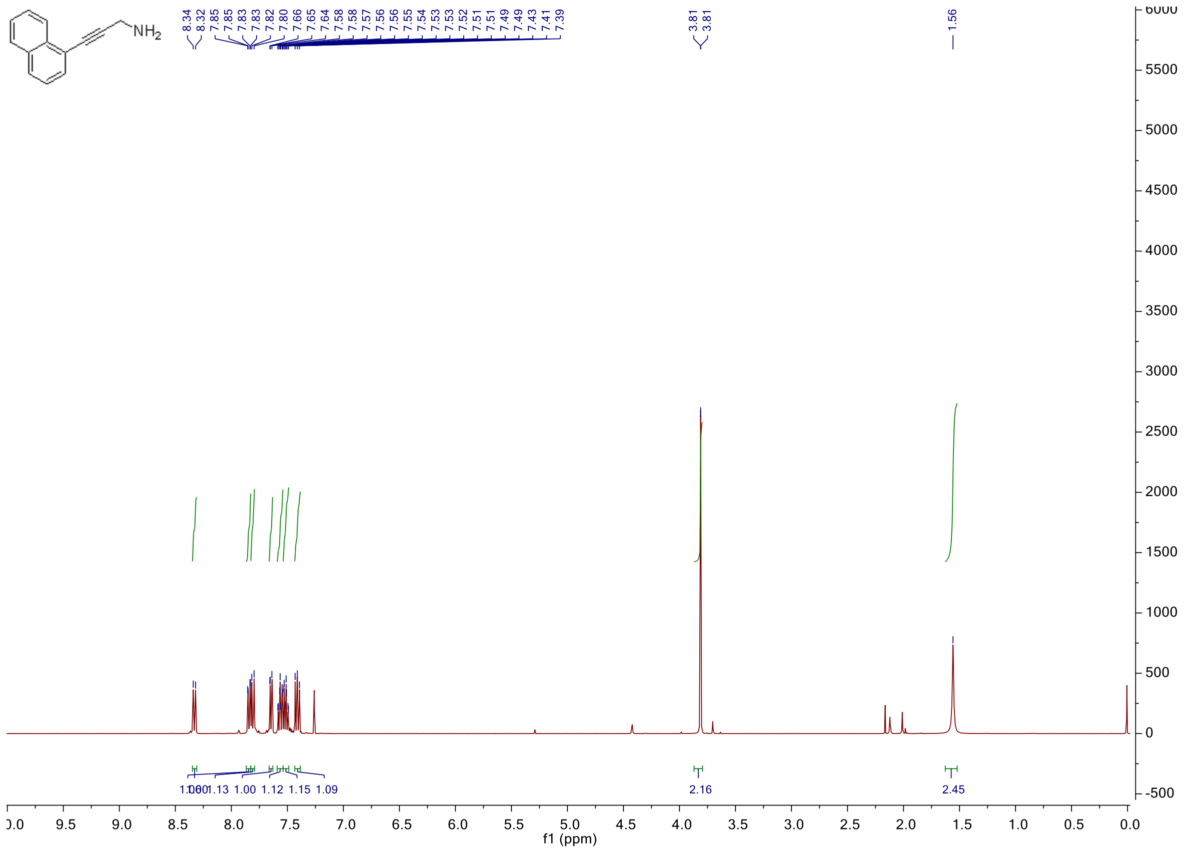

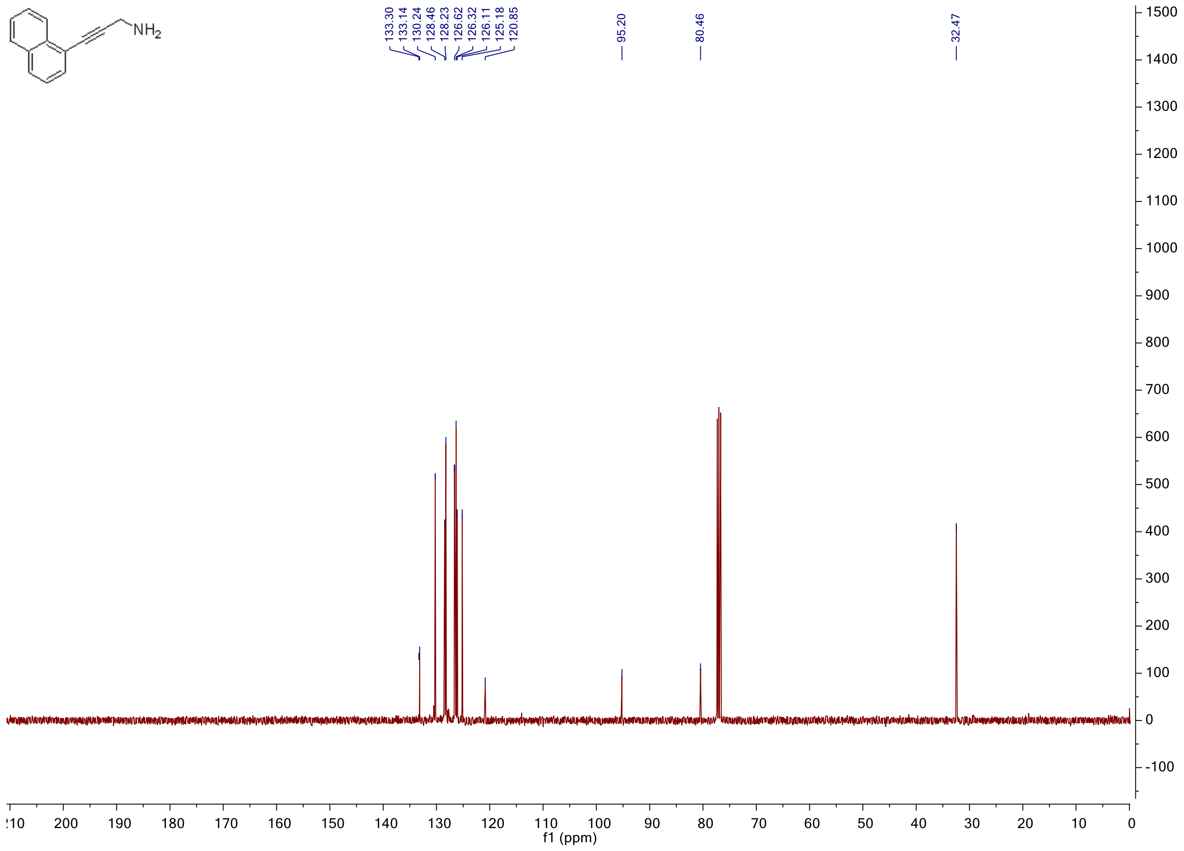

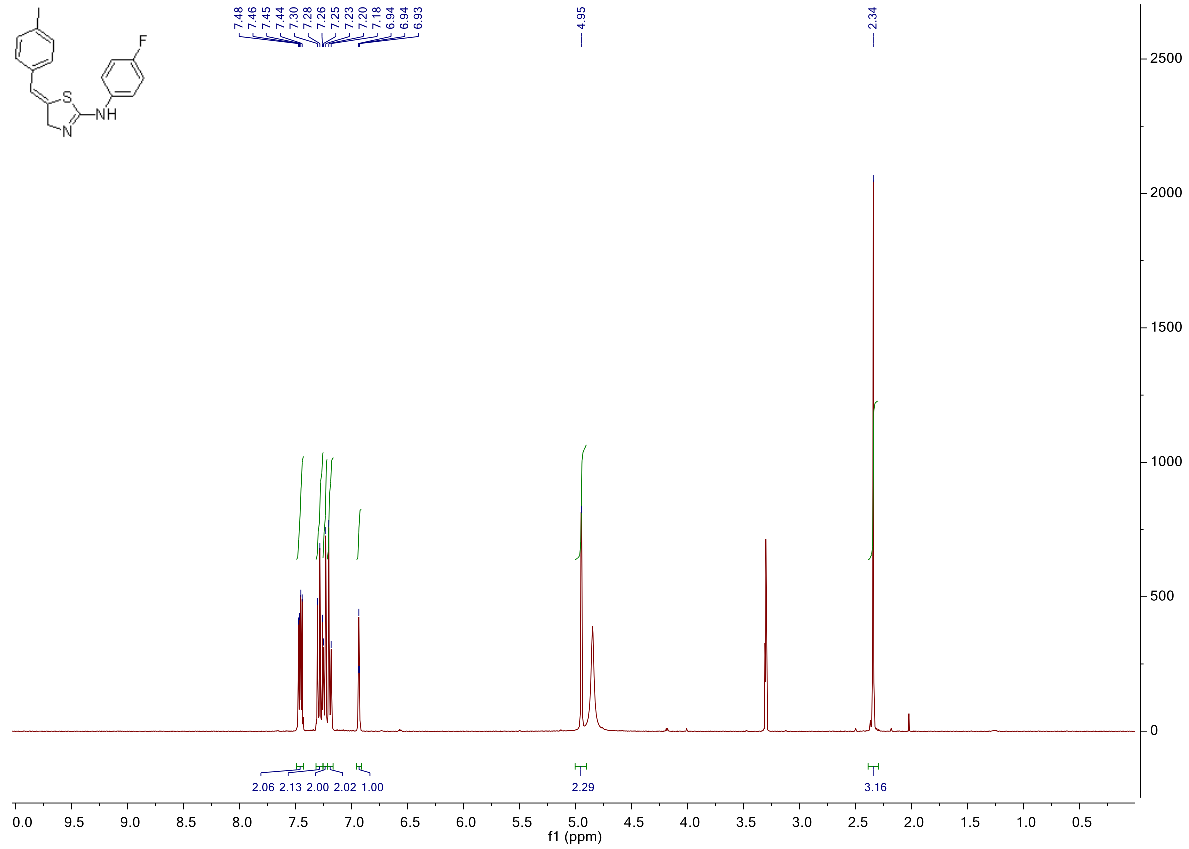

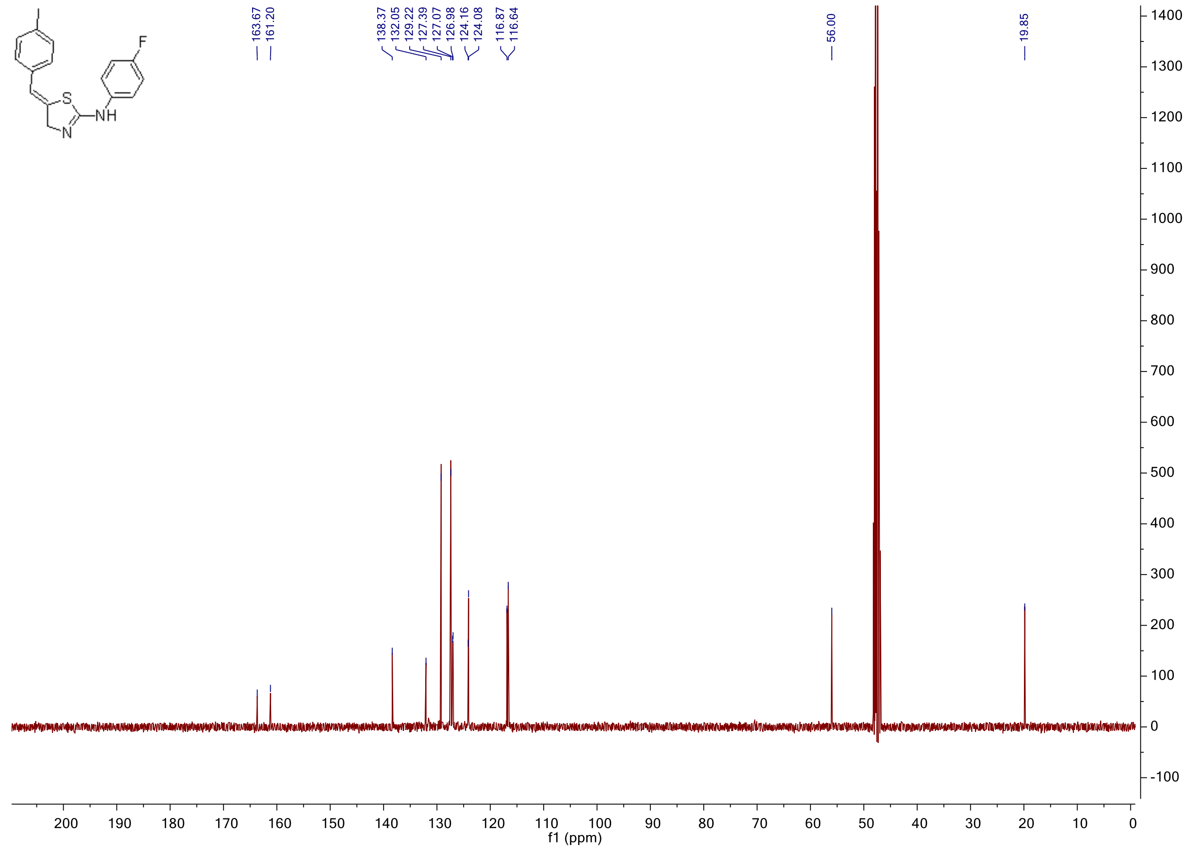

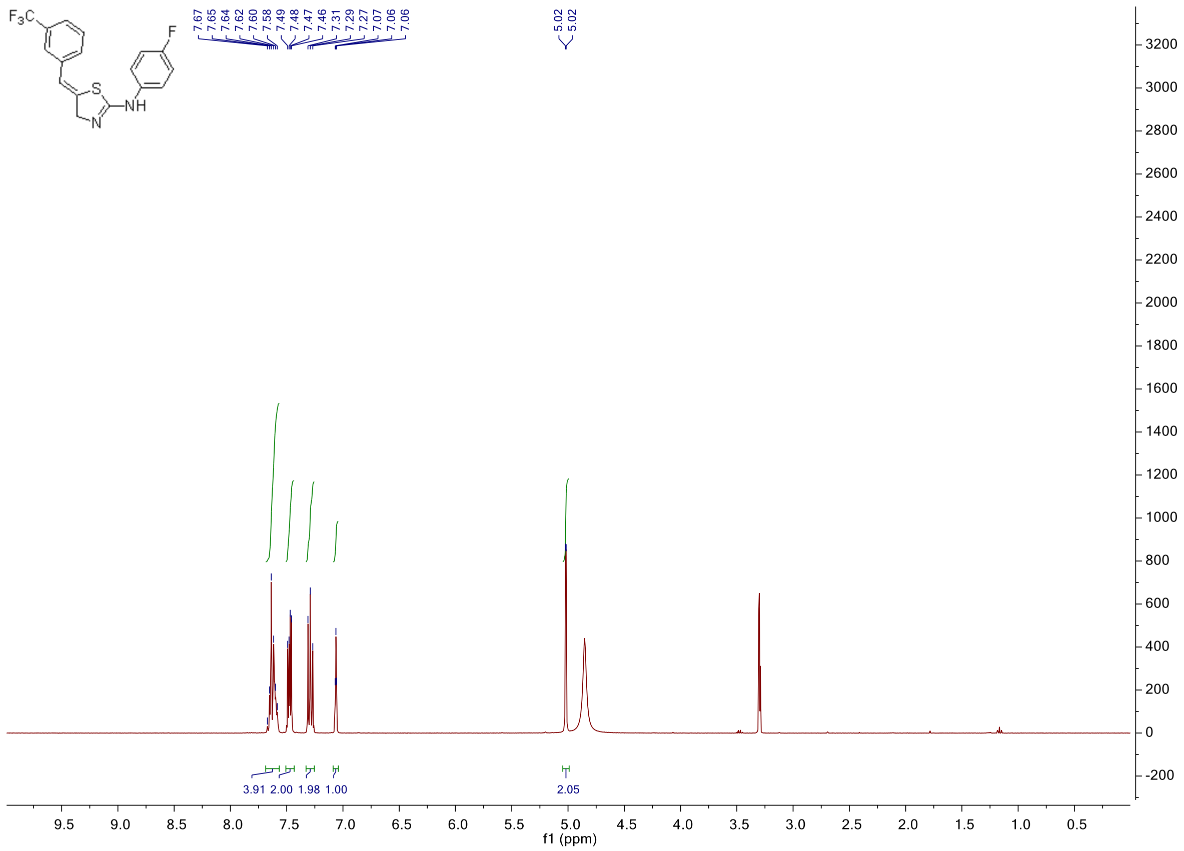

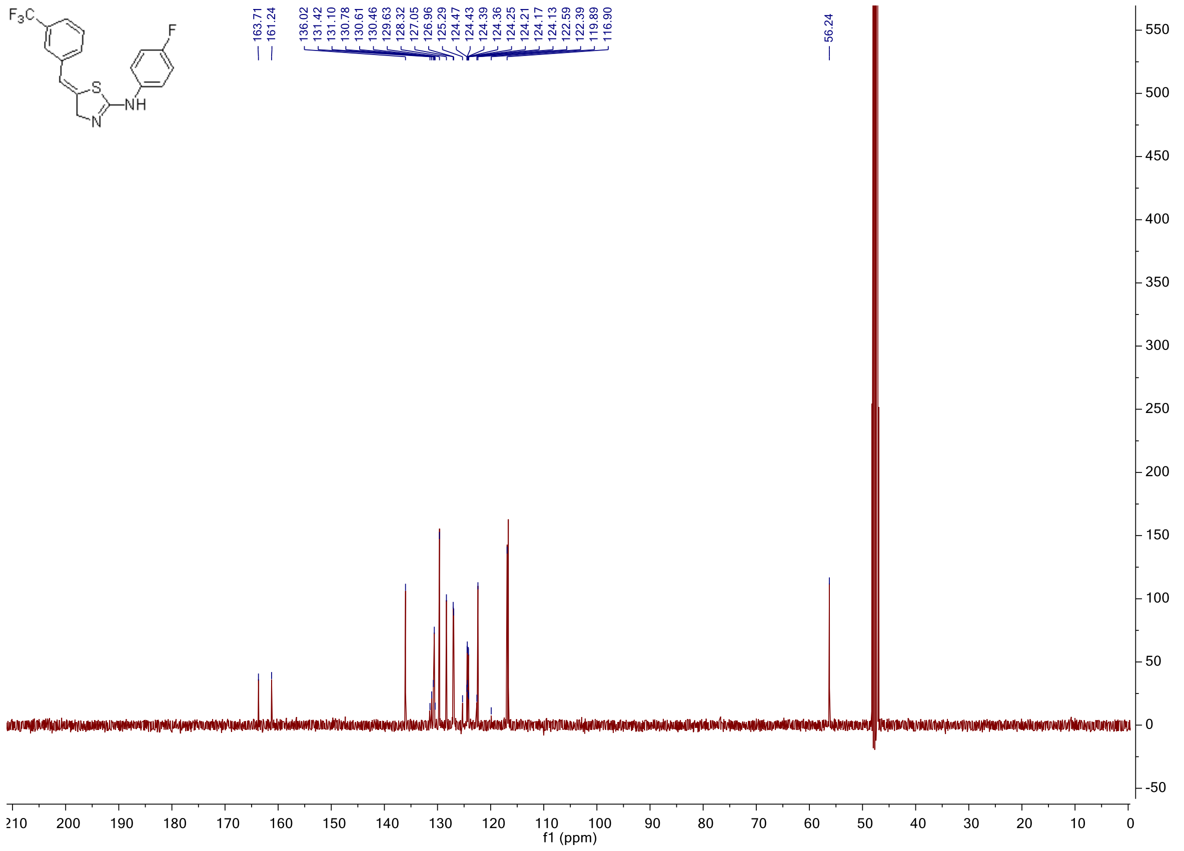

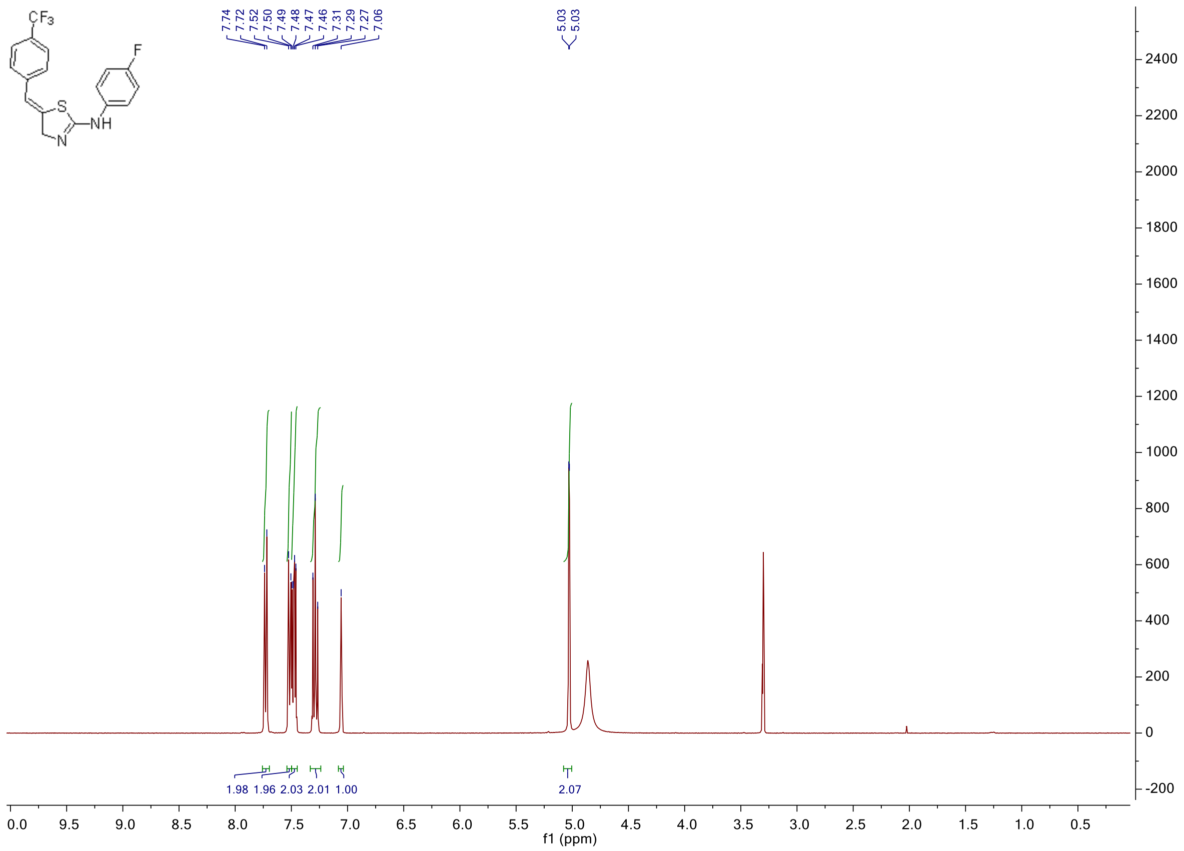

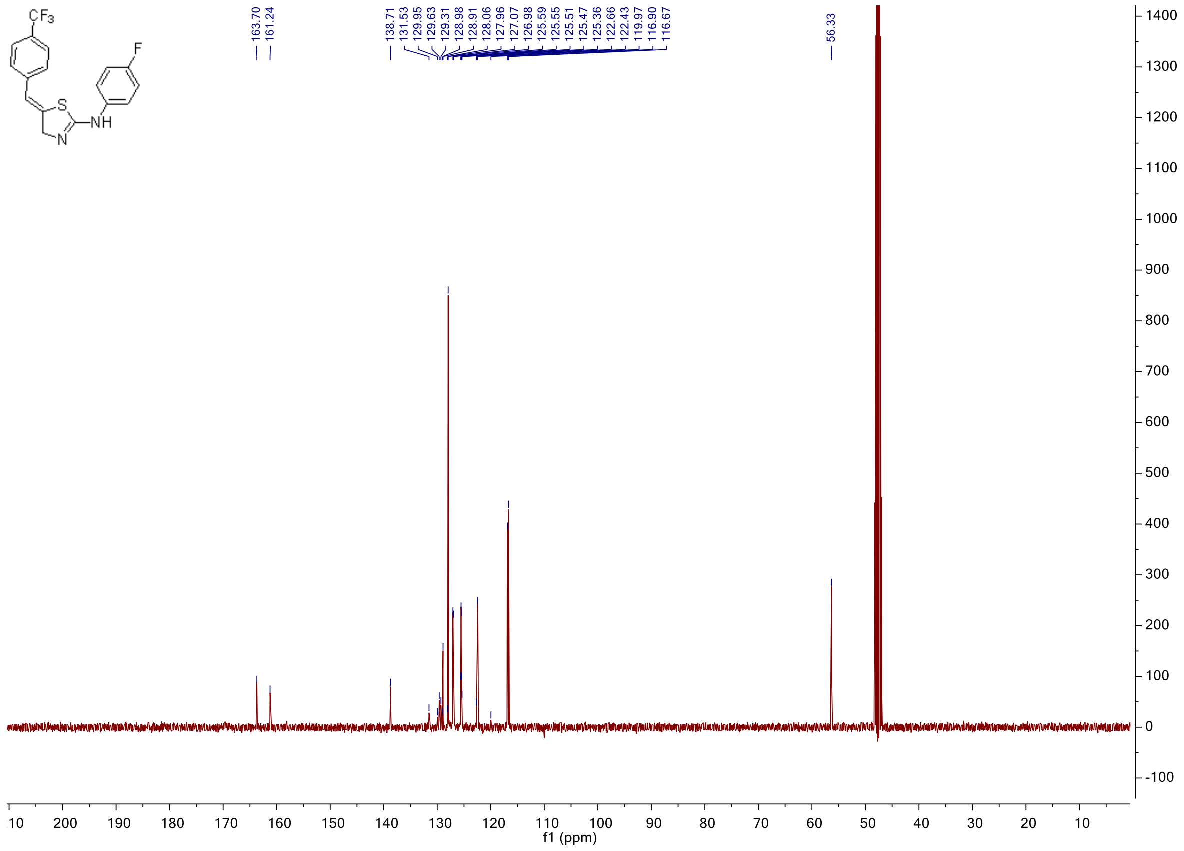

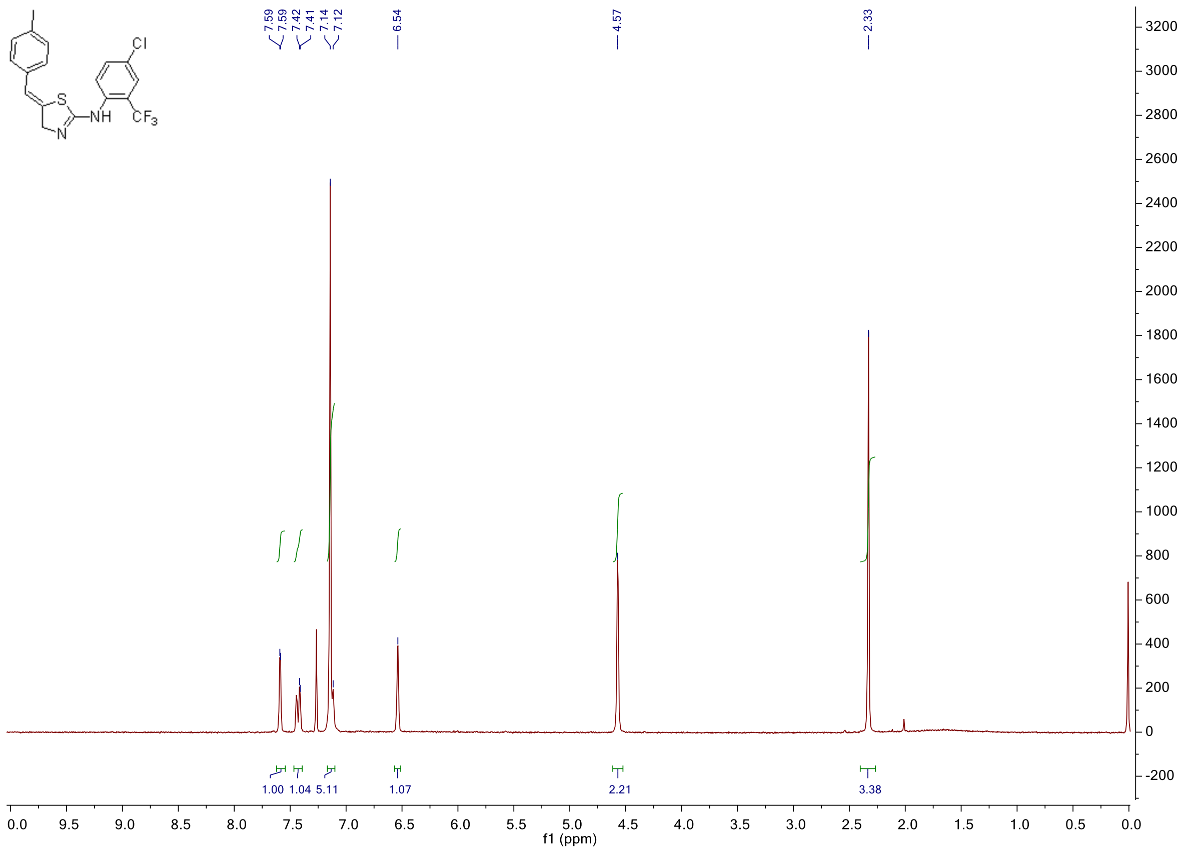

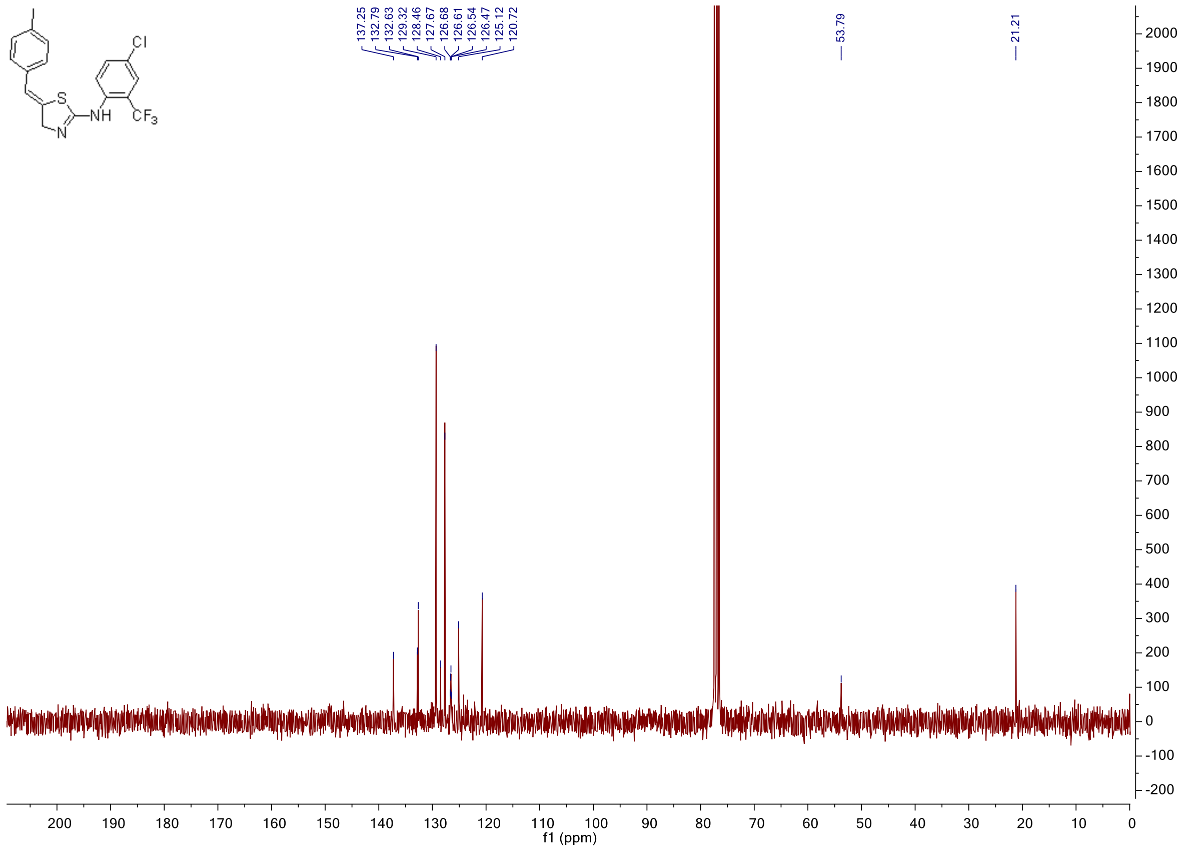

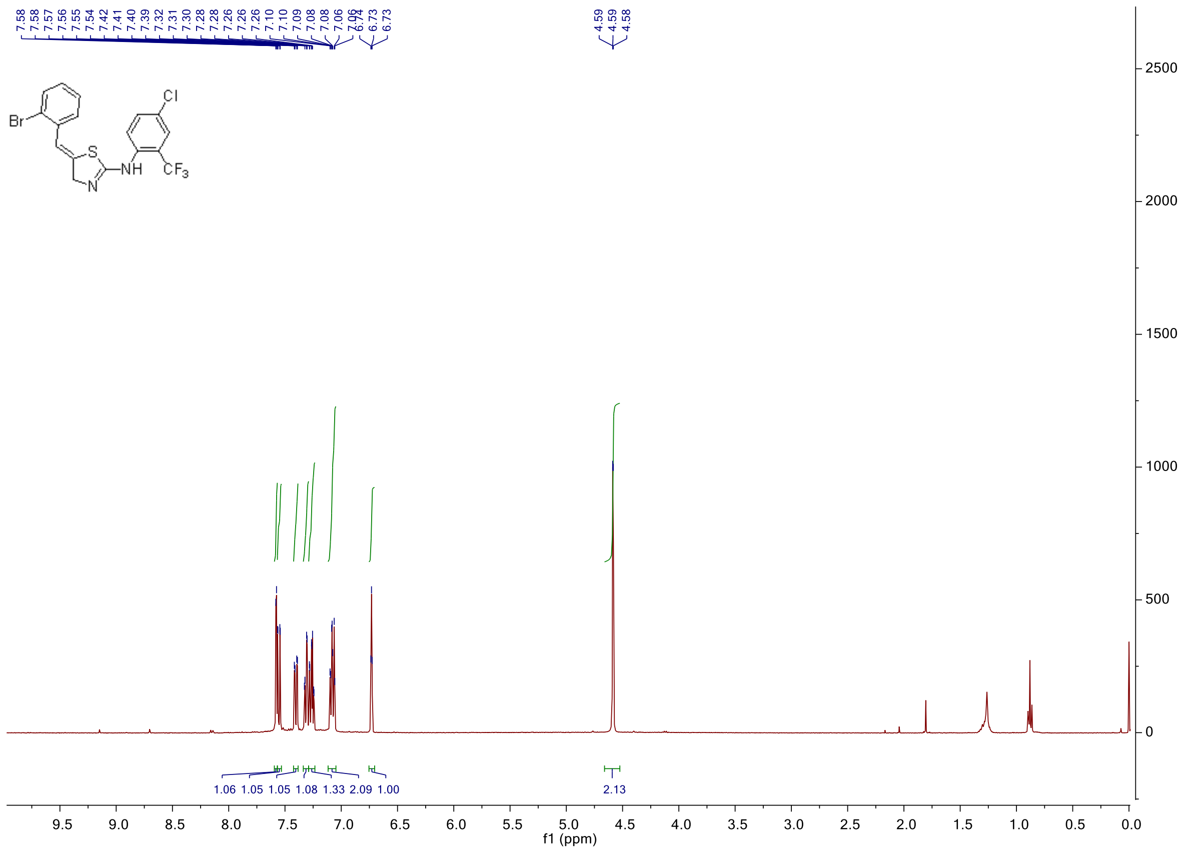
7.58 7.58 7.57 7.56 7.55 7.54 7.42 7.41 7.40 7.39 7.32 7.31 7.30 7.28 7.28 7.26 7.26 7.26 7.10 7.10 7.09 7.08 7.08 7.06 7.06 6.74 6.73 6.73
4.59 4.59 4.58
Br
Cl
S
N
NH
CF3
1.06 1.05 1.05 1.08 1.33 2.09 1.00
2.13
f1 (ppm)

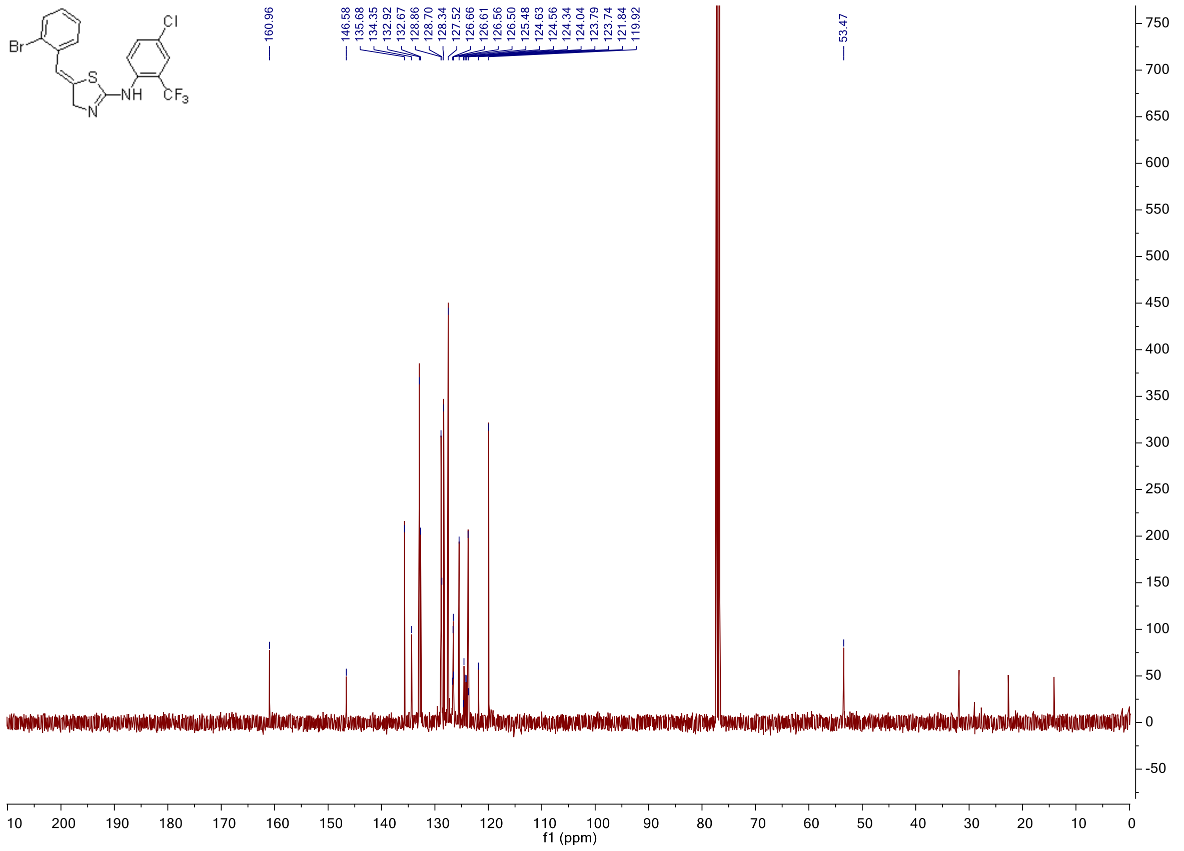
160.96
146.58 135.68 134.35 132.92 132.67 128.86 128.70 128.34 127.52 126.66 126.61 126.56 126.50 125.48 124.63 124.56 124.34 124.04 123.79 123.74 121.84 119.92
53.47
Br
Cl
S
N
NH
CF3
f1 (ppm)

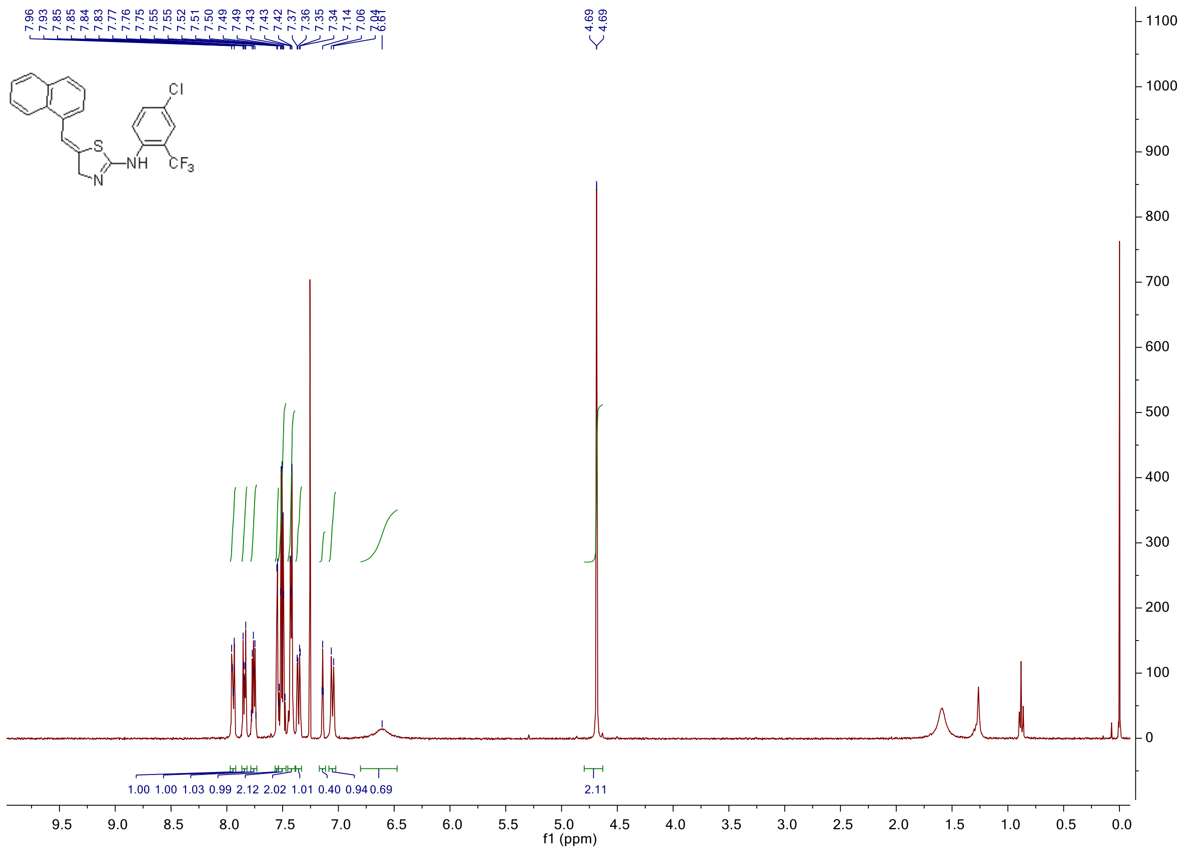

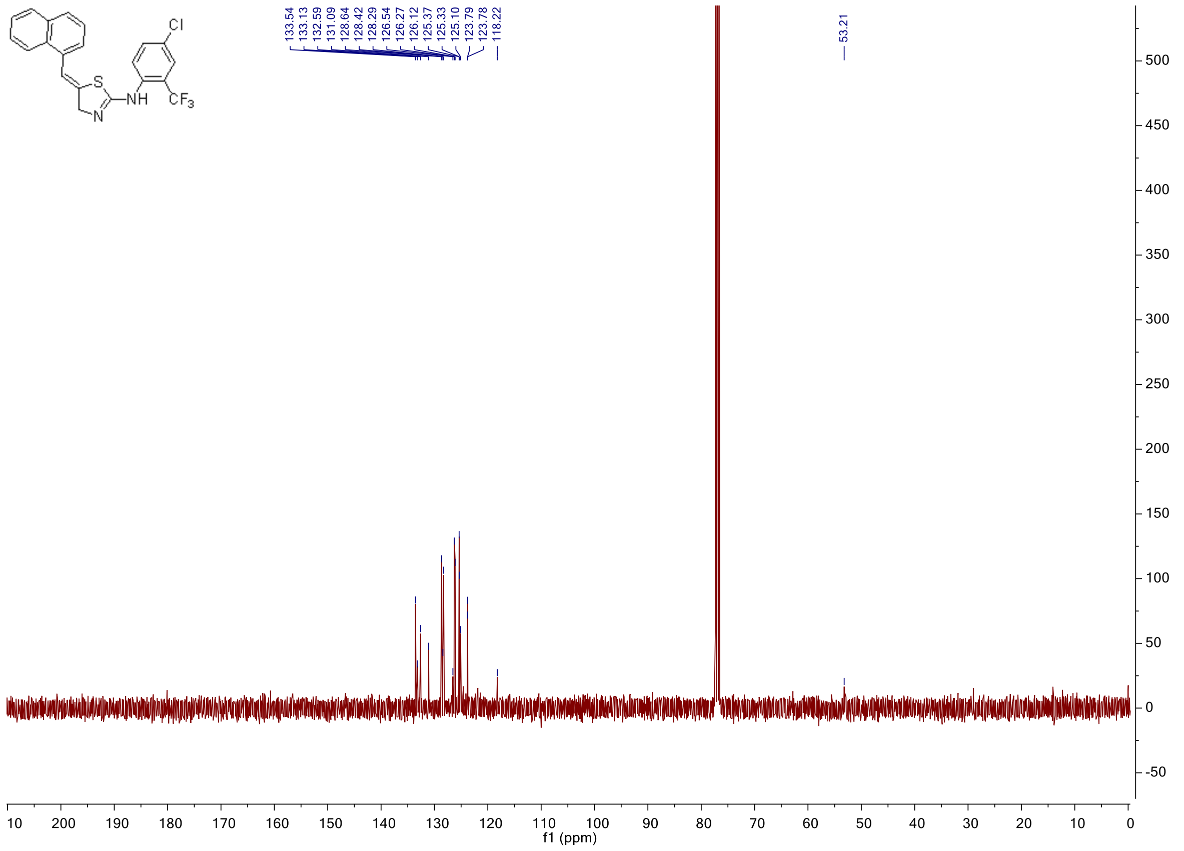

Supplement: Supplementary file 1 [file molecules-24-03658-s001.pdf]
